# Same calls, different meanings: Acoustic communication of Holocentridae

**DOI:** 10.1371/journal.pone.0312191

**Published:** 2024-11-21

**Authors:** Marine Banse, Noémie Hanssen, Justine Sabbe, David Lecchini, Terry J. Donaldson, Guillaume Iwankow, Anthony Lagant, Eric Parmentier

**Affiliations:** 1 Faculté des Sciences, Laboratoire de Morphologie Fonctionnelle et Evolutive, Universiteé de Lieège, Lieège, Belgium; 2 PSL University, EPHE-UPVD-CNRS, UAR3278 CRIOBE, Moorea, French Polynesia; 3 Laboratoire d’Excellence “CORAIL”, Perpignan, France; 4 University of Guam’s Marine Laboratory/Guam EPSCoR, UOG Station, Mangilao, Guam, United States of America; University of Windsor, CANADA

## Abstract

The literature on sound production behaviours in fish in the wild is quite sparse. In several taxa, associations between different sound types and given behaviours have been reported. In the Holocentridae, past nomenclature of the different sound types (knocks, growls, grunts, staccatos and thumps) has been confusing because it relies on the use of several terms that are not always based on fine descriptions. Our study aims to ascertain whether holocentrids can produce a variety of sounds in the wild and if these sounds are associated with specific behaviours. Additionally, we aim to determine whether sounds produced by hand-held specimens, a common methodology to record sounds in standardised conditions in fishes, could correspond to some sounds produced by free-swimming individuals in natural conditions. Our study shows that all holocentrid species are able to produce sounds in 6 behavioural contexts of both agonistic (conspecific and heterospecific chases, competition) and social signalling types (acceleration, broadcasting, body quivering), in addition to previously described mobbing towards moray eels and symbiotic interactions with cleaner wrasses. In holocentrids, acoustic communication is not only based on single calls but can also involve series of sounds of different types that are arranged randomly. The large amount of combinations within acoustical events for each behaviour, resulting from both the quantity of sounds and their diversity, supports the absence of stereotypy. This suggests that sounds are produced to reinforce visual communication during the day in this family. Our results also suggest that sounds recorded by hand-held fishes are produced naturally in the wild. Our study challenges past nomenclatures and demonstrates sound critical function in augmenting visual communication, advancing our comprehension of acoustic ecology in teleost species.

## Introduction

Communication involves a transfer of information between two or more individuals that should be beneficial to the caller at least, and eventually to the receiver [1, 2]. Up to date, almost a thousand fish taxa have been shown to be voluntary sound-producers [3]. This number is likely underestimated since many species are known to have sound-producing mechanisms, even though their sounds have not been recorded [4–6]. This underlines the critical role of acoustic communication in social interaction contexts among teleosts [7]. Vocal fish species produce sounds in a wide range of behaviours, from agonistic interactions to reproductive behaviours [8]. In some taxa such as observed in different *Dascyllus* (Pomacentridae) species, different types of sounds are stereotyped to particular behaviours [9, 10]. These *Dascyllus* species produce six different sound types associated with six different behaviours (i.e., signal jump, mating/visiting, conspecific and heterospecific chases, and conspecific and heterospecific fighting behaviours). Similarly, some species of the family Gobiidae produce two different sound types associated either with courtship or spawning [11]. In other taxa, sounds may also simply be used to reinforce a visual behaviour, as suggested in the case of *Oreochromis niloticus*, where the same type of sound can be used in various behavioural contexts [12].

Many studies investigating the vocal abilities of teleosts have used the sounds produced by hand-held (HH) fish specimens simply to highlight their vocal ability. This methodology allows the recording of sounds from different species under standardized conditions (i.e., same behavioural context of sound emission, water temperature, fish–hydrophone distance and relative position, etc.) to compare them in a reliable way [13–15]. Unfortunately, most recordings were performed in closed environments which can affect the acoustical features of sounds as a result of reverberation and resonance [16]. Besides, only few studies have investigated whether sounds produced by HH fish in controlled conditions corresponded to those produced by free-living individuals in the wild [17–20].

In holocentrids, spontaneous sound production has been reported for a wide variety of behaviours both in the wild and in laboratory conditions: when startled or handled [15, 17, 21], during territory defence [22], predator signalling and alarm calls [13, 22–24], mobbing [22, 25] and, more recently, acoustically-mediated cleaning symbiosis [26] (Table 1). One study only reported sounds during courtship activity in *Sargocentron xantherythrum* [27] but results are based on few observations from a single pair of individuals. In total, five sound types (thump, grunt, staccato, growl, knock) have been described in holocentrids.

**Table 1:** Summary of the different sound types produced by species of the family Holocentridae in the literature.

Species	Knock	Grunt	Growl	Thump	Staccato	Clicking sounds	UC
***M*. *berndti***	AB-b [24]; Und-a [24]; D-a [28]	HH/TD/AB-b [24]; Und-a [24]; D-a [28]	AB-b [24]; Und-a [24]; D-a [28]	HH-b [15]	TD/EB-b [25]; Und-a [25] D-a [29]		CS-a [26]
***M*. *violacea***	AB-b [23]	HH-b [23]; HH-b [21]	D-a [23] Und-b [23]	AB-b [23]; HH-b [15]			CS-a [26]
***M*. *pralinia***	AB-b [23]	HH-b [23]	Und-b [23]	AB-b [23]; HH-b [15]			
***M*. *amaena***	AB-b [24]; Und-a [24]; D-a [28]	TD/AB-b [24]; Und-a [24]; D-a [28]	AB-b [24]; Und-a [24]	HH-b [15]	TD/EB-b [24]; Und-a [24] D-a [28]		
***M*. *kuntee***	D-a [28]	D-a [28]; HH-b [21]		HH-b [15]	D-a [28]		CS-a [26]
***M*. *vittata***				HH-b [15]			
***M*. *jacobus***				HH-b [15]			
***M*. *hexagona***				HH-b [15]			
***M*. *adusta***				HH-b [15]			
***M*. *murdjan***				HH-b [15]			
***M*. *seychellensis***				HH-b [15]			
***H*. *rufus***		TD/AB-b [23]; Und-a [23]; HH-b [22]		HH-b [15]	TD/AB/EB/M-a [23] Und-b [22]		
***H*. *adscensionis***				S/HH-b [17] SP-a [17]; HH-b [15]			
***F*. *marianus***				HH-b [15]			
***N*. *sammara***	D-a [28]	D-a [28]; HH-b [21]	D-a [28]	HH-b [15]	D-a [28]		CS-a [26]
***N*. *aurolineatus***	D-a [28]	D-a [28]	D-a [28]		D-a [28]		
***N*. *diadema***		HH-b [21]		HH-b [15]			CS-a [26]
***N*. *microstoma***				HH-b [15]			CS-a [26]
***N*. *vexillarium***				HH-b [15]			
***N*. *argenteus***				HH-b [15]			
***N*. *coruscum***				HH-b [15]			
***N*. *punctatissimum***				HH-b [15]			
***N*. *opercularis***				HH-b [15]			
***S*. *tiere***	D-a [28]	D-a [28]	D-a [28]	HH-b [15]			
***S*. *xantherythrum***						CA-b [27]	
***S*. *caudimaculatum***				HH-b [15]	M-b [25]		HH-b [16]; CS-a [26]
***S*. *spiniferum***				HH-b [15]			CS-a [26]
***S*. *seychellense***				HH-b [15]			CS-a [26]
***S*. *rubrum***				HH-b [15]			
***S*. *praslin***				HH-b [15]			
***S*. *melanospilos***				HH-b [15]			
***S*. *dorsomaculatum***				HH-b [15]			
***S*. *tiereoides***				HH-b [15]			
***S*. *violaceum***				HH-b [15]			
***S*. *cornutum***				HH-b [15]			

Codes refer to the behaviour types (AB = aggressive behaviour; CA = courtship activity; CS = cleaning symbiosis; D = disturbance by a diver/vigilance; EB = escape behaviour; HH = handled; S = startled; SP = self-protection; M = mobbing, TD = territory defence, Und = undetermined behaviour and UC = unclassified) and the context of sound emission (a = in the wild and b = in laboratory conditions).

However, the paucity of physical descriptions (i.e., quantitative data and oscillograms) required for sound comparison, impended statistical analyses and has resulted in ambiguities. It remains uncertain whether the different authors consistently used the same terms for different sound types, or if they used different terms to potentially describe identical sounds, the different terms being mixed, in addition to the association of the different sound types with different behavioural contexts.

For instance, there is a clear confusion between the terms “grunt” and “thump”. Moulton [18] first introduced the onomatopoeia “thump” to describe pulsed sounds of 40 to 100 milliseconds (ms), produced singly at irregular intervals or in rapid volleys of 4 to 20 units in *Holocentrus adscensionis* when startled or handled in laboratory conditions. Later, Horch and Salmon [23] similarly reported that *Myripristis violacea* and *Myripristis pralinia* produced thump sounds, generally in groups of 3 to 7 in a series during aggressive behaviours. These authors also stated that *M*. *violacea* produced grunts when specimens were HH, noting that these grunts resembled thumps. Similarly, Winn *et al*. [22] considered grunts to correspond to the thumps described by Moulton [17]. Grunts were also associated with other behavioural contexts such as territorial defence against conspecifics in fish introduction experiments into tanks and chasing in response to intruders for *Holocentrus rufus*, occasionally paired with grunts [22]. Responses of *H*. *rufus* to the approach or intrusion of their territories by large heterospecific fish, as exhibited towards human observers, mainly consist in staccato calls production accompanied with dorsal fin erection [22]. The staccato call consists of a series of grunts repeated rapidly [22]. Grunt and staccato were also reported to be produced by *Myripristis berndti* and *Myripristis amaena* [24], primarily upon the introduction of a moray eel into the tank. Staccatos were mainly emitted when the eel appeared, while grunts were produced during the whole 1 minute-response recording period. This observation could indicate a certain habituation [22], similarly noted in *S*. *caudimaculatum* following the introduction of a moray-eel in their tank [25]. Growls were described as rapid series of pulsed sounds lasting from 1 to 4 seconds [24] that decreases in rate over time [23, 28]. In *M*. *berndti*, growls were reported to be produced by the aggressor in rare instances of nipping during aggressive interactions, specifically when the attacked fish did not flee [24] while *M*. *violacea* would produce growls as a response to disturbances caused by a diver [23]. Finally, knocks are short duration sounds emitted in series of variable numbers, generally up to 10, produced at irregular interval between 300 ms and 2 seconds during aggressive interactions between conspecifics, which typically consisted of larger fish briefly chasing smaller ones [23, 24]. In *M*. *berndti*, lateral displays featured two individuals in parallel alignment, either in head-to-tail or head-to-head orientations, with the fish engaging in slow circling movements. Knocking sounds were recorded when one fish broke away and was chased by the other [24].

In a more recent study, Tricas and Boyle [28] reported the production of the same sound types (knock, grunt, staccato and growl) by several holocentrid species (*Myripristis kuntee*, *M*. *berndti*, *M*. *amaena*, *Neoniphon sammara*, *Neoniphon aurolineatus* and *Sargocentron tiere*) in the field. Those were characterized as vigilance sounds since they were produced in a context of disturbance by divers or when approached by large predatory fish (e.g., carangids), meaning that the different sound types were not stereotyped to behaviours.

Confronted with a lack of precisions and uncertainties, we aim to not only revisit and clarify the primary features of the five distinct sound types previously identified in holocentrids but also seek to delineate, if possible, the behavioural contexts of their production. Consequently, this study had three main objectives: (1) to establish an ethogram of different behaviours associated with sound production during daylight in the Holocentridae, (2) to investigate whether sounds diverged with behaviours or whether the same type of sounds could be associated with different behaviours, (3) to determine if distress calls produced by HH fish could be found in natural contexts in the wild.

## Materials and methods

### Video recordings

Video recordings took place during daylight in 4 regions of the Indo-Pacific Ocean (Moorea, Guam, Seychelles and Philippines) between August 2020 and July 2022. Since our data collection involved non-invasive, simple observations of behaviours in the natural environment, and no fish were captured or handled, permits were not required for our study. Recording devices, inserted into waterproof cases, were of two types. While the first one (Spy-fish, Liège, Belgium) consisted of a modified GoPro6 (GoPro, San Mateo, CA, USA), the second one, named Cyclops, consisted of a HD video camera (Loggerhed Instruments, Sarasota, FL, USA). Both were coupled to external hydrophones HTI 96-Min (High Tech Inc., Long Beach, MS, USA, frequency range: 20 Hz– 20 kHz, sensitivity:– 164 dBV mPa^-1^). These systems were placed on the seabed at approximately 1 meter distance from caves used by holocentrids. The first system could also be fixed on a tripod so that its position could be adjusted. Experimenters would place the cameras and then leave the area to avoid any external disturbance likely to modify the behaviours of the fishes.

Sixty-four recording sessions were made, for a total duration of 77h08min. Recording effort per locality was 30 sessions in Moorea (35h33), 13 in Guam (15h56), 19 in the Seychelles (19h47) and 2 in the Philippines (5h52) (S1 Table).

### Analyses

#### Videos analysis

All behaviours associated with sounds were first marked and classified for the different holocentrid species using the DaVinci Resolve (version 1.3.2) software. All marked behaviours were then double-checked by at least two researchers to identify the species, confirm caller identifications and categorize the behaviour type. This approach by several observers has the great advantage of increasing the reliability of the observations. Doubtful observations were not included in the study.

#### Studied species

Nine holocentrid species belonging to the family’s three most diverse and abundant genera (*Myripristis*, *Sargocentron* and *Neoniphon)* were recorded during this study: *M*. *kuntee*, *M*. *berndti*, *M*. *violacea*, *Neoniphon diadema*, *Neoniphon sammara*, *Neoniphon argenteus*, *Neoniphon microstoma*, *Sargocentron spiniferum*, *Sargocentron seychellense*. From these nine species, the two with the highest numbers of recorded acoustical events among each genus were selected for analysis (S2 Table): *M*. *kuntee*, *M*. *violacea*, *N*. *diadema*, *N*. *sammara*, *S*. *spiniferum*, *S*. *seychellense*.

#### Sound analysis

**Fig 1:**
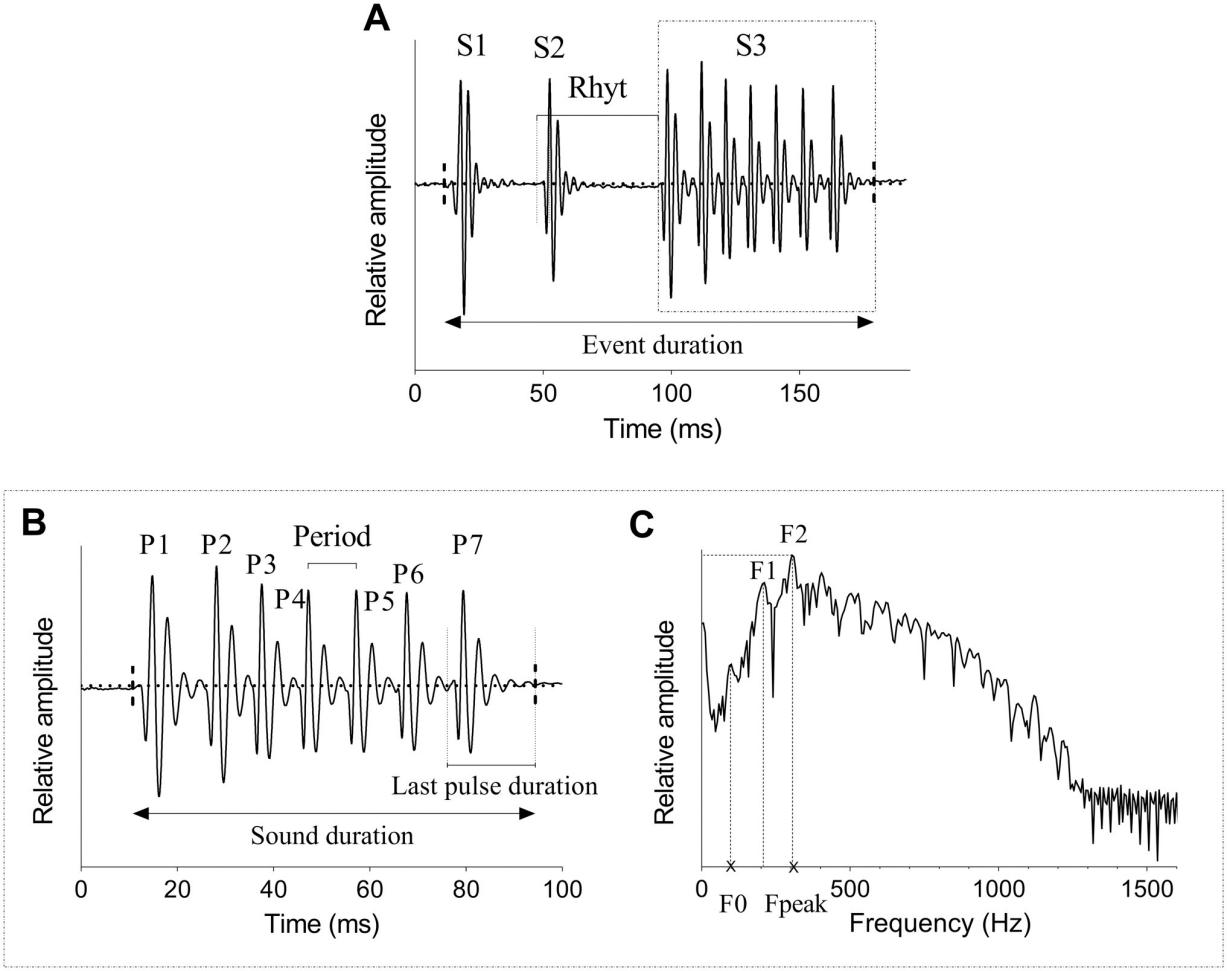
Oscillograms of an example acoustical event (A) with a detailed view of sound S3 (B) and power spectrum of sound S3 (C) in *Neoniphon sammara*. S = sounds; rhyt = rhythm; P = pulses; F = harmonics; F0 = fundamental frequency; Fpeak = dominant frequency.

Soundtracks were extracted from the videos analysed in DaVinci Resolve and acoustical events isolated from these soundtracks. An acoustical event refers to the production of one or several sounds produced by an individual during a behaviour. Events were first band-pass filtered (50–1000 Hz) to reduce background noise. Both acoustical events and sounds composing these events were then manually investigated using the software Avisoft-SAS Lab Pro 5.2.13 (Avisoft Bioacoustics, Glienicke, Germany). Three acoustical parameters were measured on the events (Fig 1): (1) duration of the event (ms), (2) number of sounds composing the event, (3) rhythm (or sound period, measured as the time interval between the beginning of two consecutive sounds, ms). Six additional acoustical variables were measured on the sounds themselves (Fig 1): (4) sound duration (ms), (5) number of pulses in the sound, (6) pulse periods (measured as the peak-to-peak intervals between two consecutives pulses, ms), (7) duration of the last pulse in the sound (ms) based on oscillograms (Fig 1A and 1B), (8) fundamental frequency (Hz) and (9) dominant frequency (defined as the frequency with the highest energy, Hz) of the sound based on power spectra (Fig 1C).

**Fig 2:**
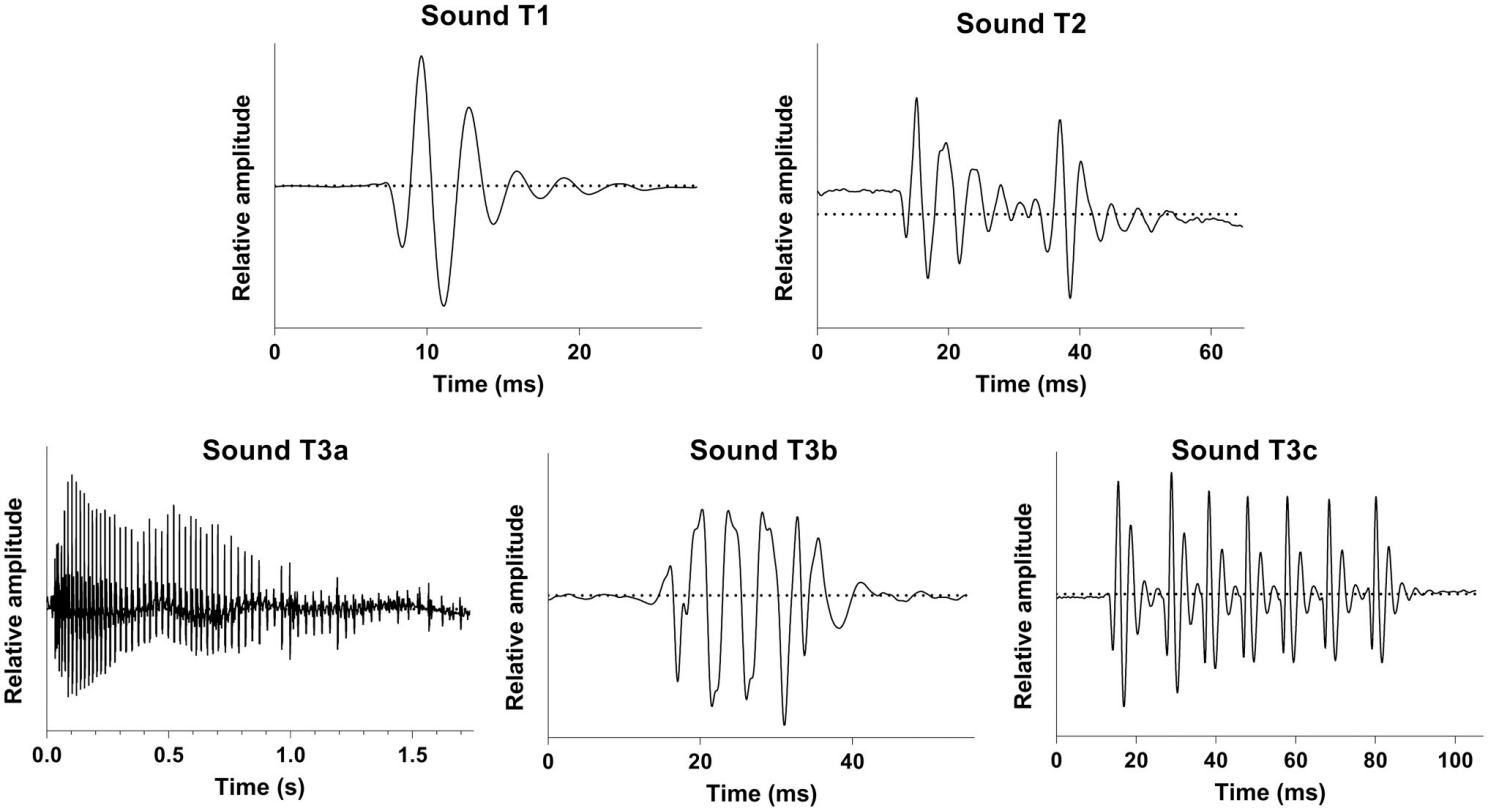
Oscillograms of the 5 types of sounds produced by the Holocentridae in the wild.

Sounds were classified into different categories based on their number of pulses (Fig 2): (T1) single-pulse sounds, (T2) sounds composed of 2 pulses and (T3) sounds composed of more than 2 pulses. In order to investigate if the sounds produced by HH fish in standardized conditions at sea corresponded to the sounds produced by free-swimming fish in the wild, acoustical data from Banse *et al*. [15] were used in this study for comparison. Because we observed significant variations in the acoustic parameters of T3 sounds and noted on oscillograms that some of these sounds bear striking similarities to HH sounds, preliminary analyses were conducted to identify the common acoustic characteristics that would allow HH sounds to be associated with certain T3 sounds. This approach to categorize T3 sounds resulted in the creation of three subcategories, based on both the sound duration and the shortest pulse period within the sound (Fig 2): (T3a) sounds lasting more than 150 ms with increasing pulse period towards the end of the call, (T3b) sounds lasting less than 150 ms having their smallest pulse period < the smallest pulse period in sounds produced by HH fish, and (T3c) sounds lasting less than 150 ms having their smallest pulse period ≥ the smallest pulse period of sounds produced by HH fish. Note that the minimum pulse period of sounds produced by HH fish was obtained for each the species and consequently differed between species. Besides, this distinction between T3b and T3c sounds corresponded to an observable character related to the pulse period. In T3b sounds, individual pulses were mostly made of a single peak and could therefore only be identified by their initial peak, whereas T3c sounds pulses were distinctly recognizable due to their multiple peak nature (Fig 2).

#### Statistical analyses

All analyses were performed in RStudio version 2023.9.0.463 [29]. Descriptive statistics were calculated for each temporal and spectral property of the acoustical signals for both events and sounds within the events, for each species. In the results, “n” refers to the total number of analysed sounds and “N” to the number of events; n = y, N = x means that the analysis was made on y sounds coming from x events. We excluded from the analyses (1) behaviours whose number of acoustical events was < 5 for each species and (2) two competition events that were extremely long with respect to all other events for *N*. *sammara* (S2 Table). Furthermore, for each behaviour and species, we excluded from the statistical comparisons of acoustical features related to sounds the sound types that did not have at least 5 observations (S3 Table).

*Univariate statistics*. Univariate statistics were first carried out to investigate (1) variations in acoustical features of both events and sounds within these events between the different behaviours within each species and (2) whether sounds produced by HH fish correspond to the T3c sounds produced by free-swimming individuals in the wild for 3 species (*N*. *sammara*, *S*. *spiniferum* and *M*. *violacea*). These species were selected for the latter comparison because the number of T3c sounds recorded was sufficient to perform statistical comparisons with HH sounds, while only 4 T3c sounds were recorded for *N*. *diadema* and none for *S*. *seychellense* and *M*. *kuntee*. The normality of the data and the homoscedasticity of the variances were first assessed to determine if parametric or non-parametric tests should be used to perform the statistical analyses, respectively using Shapiro-Wilk tests and Bartlett’s tests, with a significance level *p* < 0.05. Data were log- or square root-transformed if it allowed to meet both criteria before the analyses. T-tests, Mann-Whitney-Wilcoxon tests, ANOVA followed by post-hoc Tukey’s tests with a significance level of *p* < 0.05 or Kruskal-Wallis tests followed by post-hoc Dunn’s tests with Benjamini-Hochberg correction with a significance level of *p* < 0.025 (α/2, since we used the parameter altp = FALSE in the dunn.test function) were then chosen accordingly and performed on the data.

*Multivariate statistics*. Principal component analyses (PCA) were additionally performed on HH and T3c sounds for three species (*N*. *sammara*, *S*. *spiniferum* and *M*. *violacea*). For the interpretation of PCA results, we considered the number of factors equivalent to the number of eigenvalues greater than 1.0. Convex hulls (CH) were built for each group in the different scatterplots. 3D convex hulls were represented in the 3D interactive scatterplots using the cxhull function of the cxhull package. The 3D interactive scatterplot created using the first three principal components (PCs) from the PCA can be found in S1 Data. By clicking on the legend components, one can then decide which element to visualize or hide in this interactive 3D environment.

## Results

### Behaviours associated with sound production

The holocentrids examined in this study could produce sounds during the execution of at least 6 different behaviours (Table 2): (1) acceleration, (2) conspecific chase, (3), heterospecific chase, (4) competition, (5) broadcasting and (6) body quivering. A total of 1382 sonic events were recorded for all behaviours for the 6 studied species (S2 Table). The number of recorded acoustical events varied between species, most likely due to specimen abundance. Behaviours related to reproduction were not observed. The six behaviours could be classified into two main groups: agonistic and social signalling. A total of 65.6% of behaviours corresponded to agonistic interactions towards conspecifics and heterospecifics, encompassing aggressive interactions and competition characterized by parallel swimming movements. In social signalling behaviours, fish signal their presence without the message appearing to be specifically directed at a precise recipient. Social signalling behaviours included acceleration, broadcasting and body quivering. They constituted 34.4% of the observed behaviours.

**Table 2:** Ethogram of the different behaviours associated with sound production performed by species of the family Holocentridae in the wild.

Behaviour	Abbreviation	Description	Behaviour type	Supplementary material
**Acceleration**	Acc	A solo individual abruptly increases its swimming speed	Social signalling	S1 Movie
**Conspecific chase**	Chase_cs	An individual chases a conspecific	Agonistic	S2 Movie
**Heterospecific chase**	Chase_hs	An individual chases an heterospecific	Agonistic	S3 Movie
**Competition**	Cp	Two conspecific fish swim parallel	Agonistic	S4 Movie
**Broadcasting**	BC	An individual highlights its presence (e.g., dorsal fin erection, moving or turning, body agitation)	Social signalling	S5 Movie
**Body quivering**	BQ	Body quivering of the individual without fish displacement	Social signalling	S6 Movie

Videos of the different behaviours are available in S1–S6 Movies.

**Fig 3:**
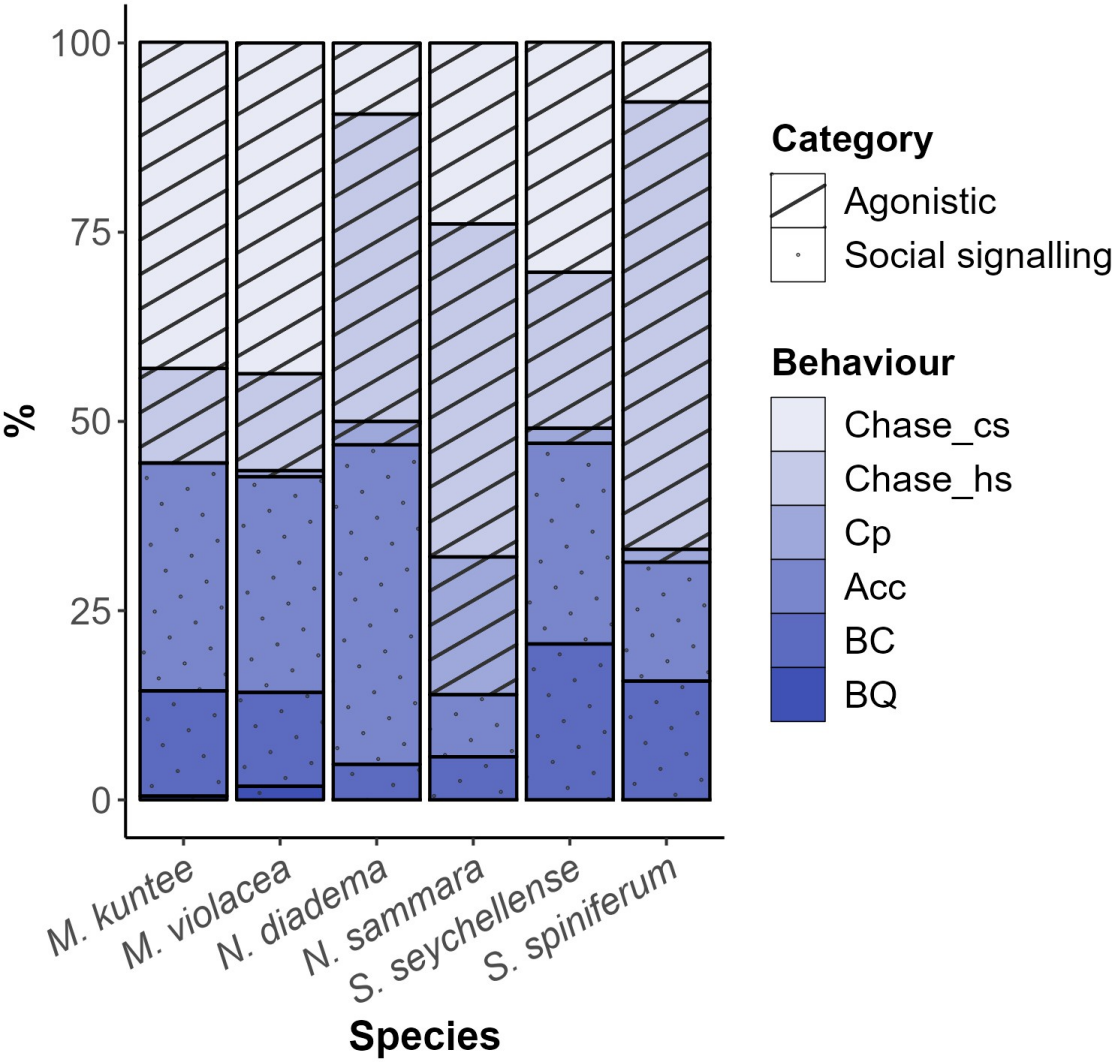
Stacked bar plot representing the percentage of each behaviour associated with sound production performed by the different Holocentridae species investigated. Chase_cs = conspecific chase; Chase_hs = heterospecific chase; Cp = competition; Acc = acceleration, BC = broadcasting, BQ = body quivering.

*Myripristis kuntee* and *N*. *diadema* did not display competition, and body quivering was not observed in *N*. *sammara*, *S*. *seychellense*, and *S*. *spiniferum* (Fig 3).

### Intraspecific comparison between behavioural events

For each species, univariate statistical analyses were performed to compare the three acoustical variables measured on acoustical events between the different behaviours (Fig 1). For all species, there was no variation in the rhythm of sound production across the different behaviours (Kruskal-Wallis tests, *p* < 0.05; Table 3; S4 Table), except in *S*. *spiniferum* where the rhythm of the conspecific chase behaviour was more than twice as high as the rhythm of the acceleration and heterospecific chase behaviour (Tukey’s test following ANOVA, *p* < 0.05; S5 and S6 Tables). Although differences are observed for the two additional acoustic features describing the events (event duration and number of sounds composing the event) between behaviours, these differences are not consistent across species (S4–S7 Tables). Therefore, detailed results for each species will be given in S1 Text. In a general way, social signalling behaviours are shorter and composed of fewer sounds than agonistic behaviours.

**Table 3:** Summary of mean ± sd and [min–max values], calculated for each behaviour and species, for the different acoustical variables of the acoustical events.

Species	Behaviour	N	Event duration (ms)	n	Rhythm (ms)
*M*. *kuntee*	Acc	65	221.6 ± 272.1 [3.9–1434]	2.4 ± 1.6 [1–7]	152.9 ± 123.1 [9.1–537]
	Chase_cs	93	315.6 ± 409.6 [5.5–2345]	3 ± 2.2 [1–13]	140.8 ± 123.5 [7–657]
	Chase_hs	27	271.3 ± 267.2 [5.4–972]	3 ± 1.9 [1–8]	128.7 ± 97.7 [7.6–475.9]
	BC	30	117.1 ± 122.6 [5.4–403.5]	2 ± 1 [1–5]	111.2 ± 84.1 [9.6–337]
*M*. *violacea*	Acc	142	124.1 ± 177.4 [3–1232]	2 ± 1.6 [1–11]	105.8 ± 84.3 [6.2–459]
	Chase_cs	218	315.5 ± 327.3 [5.3–1746]	3.3 ± 3 [1–29]	129 ± 107.9 [9.9–872]
	Chase_hs	64	327 ± 356.3 [5.6–2071]	3.3 ± 2.5 [1–12]	133.1 ± 124.9 [13.1–698]
	BC	62	157.3 ± 219.5 [7.2–1223.2]	2 ± 1.3 [1–6]	126.4 ± 90.6 [18.3–400]
	BQ	9	134.7 ± 212.1 [8.6–543.7]	1.7 ± 0.7 [1–3]	183.3 ± 224.5 [31.3–525]
*N*. *diadema*	Acc	27	61.4 ± 72 [10.3–306.7]	1.3 ± 0.5 [1–3]	110 ± 94.8 [18.9–296]
	Chase_cs	6	1946.4 ± 1225.8 [498.5–3703]	13.3 ± 8 [4–26]	156.1 ± 76.8 [37.8–455.2]
	Chase_hs	26	1121.1 ± 1138.8 [12.1–4495]	8.2 ± 8.3 [1–37]	152.5 ± 113.9 [14.7–1055]
*N*. *sammara*	Acc	33	225.1 ± 300.6 [10–1371]	2.3 ± 1.6 [1–7]	165.2 ± 125.8 [15.9–492.8]
	Chase_cs	96	422.5 ± 557.9 [9.7–3907]	3.1 ± 2.2 [1–12]	194.1 ± 162.9 [18–1257]
	Chase_hs	177	381.1 ± 459.8 [6.9–2941]	2.9 ± 2.2 [1–15]	187.5 ± 137.3 [18.6–840]
	Cp	71	1191.6 ± 1206 [11.6–5440]	6.9 ± 6 [1–39]	201.4 ± 215.4 [17.5–1846]
	BC	23	154.4 ± 218 [9.2–825.9]	1.7 ± 1.1 [1–6]	221.3 ± 221 [25.3–815.2]
*S*. *seychellense*	Acc	27	289.2 ± 595.3 [3.7–3141]	2.1 ± 1.1 [1–5]	246 ± 379.9 [29.1–1843]
	Chase_cs	31	592.7 ± 1072.7 [10–4262]	3.3 ± 3.7 [1–17]	247.5 ± 308.2 [15.7–1706]
	Chase_hs	21	405.2 ± 752.2 [11.1–3457]	3.2 ± 3.9 [1–18]	174.3 ± 127.2 [31.2–485.4]
	BC	21	128.4 ± 162.5 [4.6–511]	1.6 ± 0.9 [1–4]	181.6 ± 150.6 [48.8–490.8]
*S*. *spiniferum*	Acc	18	226.5 ± 223.3 [16.7–760.1]	2.5 ± 1.6 [1–6]	127.2 ± 90.4 [17.6–377.8]
	Chase_cs	9	982.9 ± 1274.1 [13.9–3761]	4.7 ± 4.6 [1–13]	260.5 ± 249.3 [57.3–1270]
	Chase_hs	68	404.1 ± 555.4 [9.2–2872]	4.2 ± 4.7 [1–26]	117.9 ± 139.4 [12.2–1592]
	BC	18	151.4 ± 185.4 [8.7–689.5]	1.7 ± 1.4 [1–7]	164.9 ± 187.5 [29.2–672.9]

N = number of acoustical events.

### Sounds composing the acoustical events

#### Different sound types

Since each acoustical event consisted of either a single sound or a series of sounds, it became essential to determine if there was an organizational pattern or code associated with behaviours or species. Identifying the potential units of this code was therefore crucial. This involved the classification of sounds into three main types (Fig 2): single-pulse sounds (T1), sounds composed of 2 pulses (T2), and sounds composed of more than 2 pulses (T3), themselves subdivided into three groups (T3a, T3b and T3c).

Due to the high similarity in pulse shapes on the oscillograms, we hypothesize that these pulses are produced using the same motor pattern, simply expressed at different periodicities. In total, 4345 sounds that composed the 1382 acoustical events were selected for the analyses (S3 Table): 3786 sounds T1, 262 sounds T2 and 297 sounds T3 (10 sounds T3a, 193 sounds T3b and 94 sounds T3c). Sounds T1 were found in 1265 events, whereas sounds T2 and T3 were found in 192 and 210 events, respectively. Finally, T3a sounds were found in only 9 events while T3b and T3c sounds composed 139 and 69 events, respectively. For all behaviours and species, acoustical events were mostly composed of T1 sounds (min. 54.3%—max. 100%) (S3 Table).

**Fig 4:**
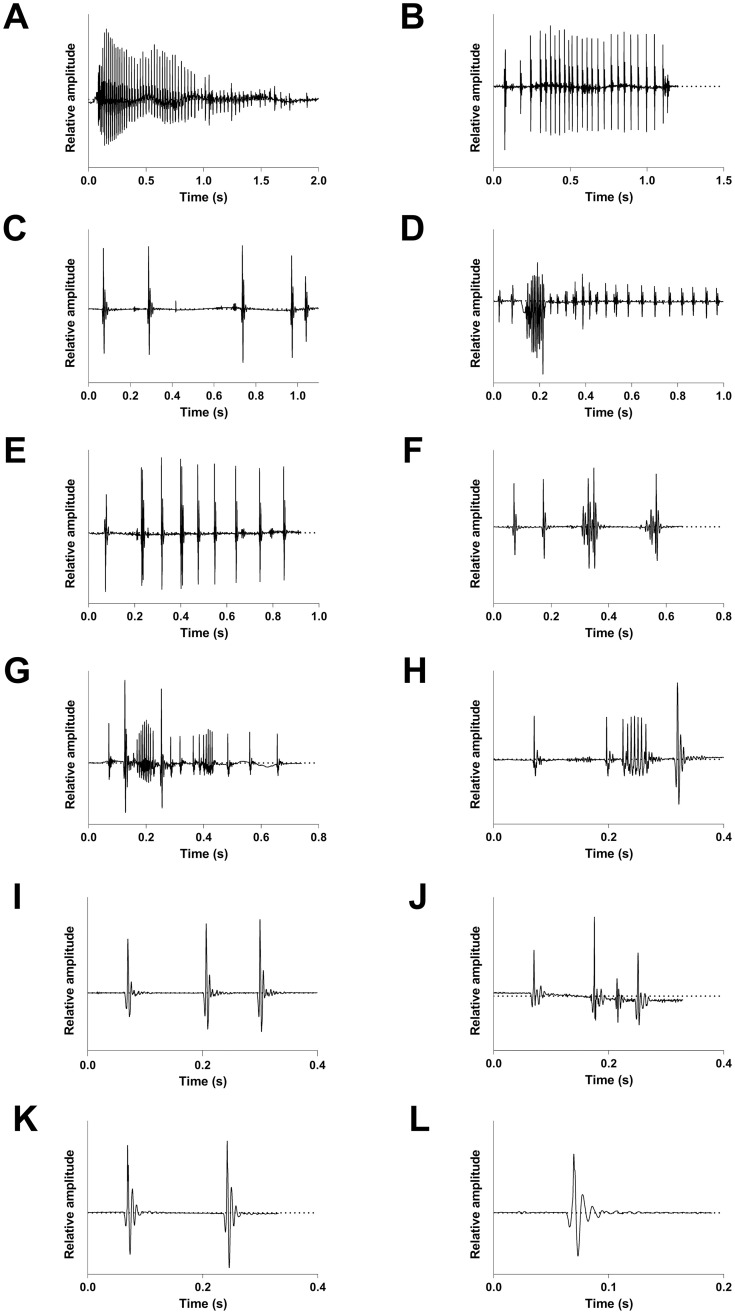
Oscillograms of 12 acoustical events related to heterospecific chases produced by *S*. *spiniferum*.

Organizing sounds by types and observing their arrangement within events could have evidenced a kind of phraseology linked to either species or behaviours. For all species however, our results indicate that the different sound types (T1, T2 and T3) composed the acoustical events of most behaviours, with no specific sound type uniquely linked to a particular behaviour (S3 Table). Moreover, the events did not show a fixed number of sounds nor a stereotyped combination of the different sound types in terms of order or periodicity, reinforcing the absence of association between acoustical signals and specific behaviours or species. Similarly, several behaviours were composed of sound types T3a, T3b and T3c in all species (S3 Table). Consequently, there is a huge number of sound type combinations associated to each behaviour for each species (S8 Table). As an example, the events associated to the heterospecific chase in *S*. *spiniferum* (N = 68) provided 26 different acoustical combinations (Fig 4; S8 Table). These acoustical combinations seem to be rather random instead of corresponding to a stereotyped motor pattern. Furthermore, we noted that between 55.6% and 100% of the acoustical events begin with a single-pulse sound for all behaviours and species (S9 Table), which is consistent with the very large proportion of sound type T1 (> 50%) found in the events.

#### Intraspecific comparison of sounds between behavioural events

Although the event organization was not stereotyped, we sought whether each sound type (T1, T2, T3a, T3b, T3c) could differ between behaviours. However, only sporadic differences were observed for the different sound types between behaviours, without general tendencies across species (Tables 4–8; S10–S23 Tables). In other words, each sound type usually shares similar acoustical features across behaviours for each species. The analysis clearly supports that there is no specific type of sound (T1, T2, T3a, T3b or T3c) that correlates with a behaviour for all species. Considering the large number of behaviours and sound types investigated, the likelihood of identifying some differences was significant. However, the comparison of the results across the different species does not support a biological meaning. Indeed, when differences do exist between behaviours for a sound type, they do not necessarily relate to the same features and seem rather random, which indicates that the features composing a sound type do not carry biological significance. As a practical example, it cannot be claimed that a T1 sound produced by a species during a given behaviour has different features than a T1 sound produced during another behaviour. However, in the interest of intellectual thoroughness and to be completely transparent with our analysis, all features’ measurements and statistical comparisons for the different sound types and species are detailed in Tables 4–8 and S10–S23 Tables, along with a description for each species in S2 Text.

**Table 4:** Summary of mean ± sd and [min–max values], calculated for each behaviour and species, for the different acoustical variables of sounds T1 (n = 3786).

Species	Behaviour	n	Sound duration (ms)	Fpeak (Hz)
*M*. *kuntee*	Acc	130	8.6 ± 4.4 [3.1–21]	401 ± 210 [119–919]
	Chase_cs	193	7.9 ± 2.7 [2.9–15.1]	340 ± 174 [164–890]
	Chase_hs	64	8 ± 2.9 [2.6–17.1]	376 ± 198 [158–896]
	BC	41	7.6 ± 2.9 [3.3–15.1]	410 ± 172 [164–861]
*M*. *violacea*	Acc	211	13.5 ± 5 [3–28.3]	282 ± 106 [99–685]
	Chase_cs	607	15.2 ± 5.6 [3.1–50.6]	226 ± 147 [70–990]
	Chase_hs	172	12.7 ± 4.6 [5–27.8]	259 ± 136 [64–822]
	BC	118	13.5 ± 5.6 [4.7–41.4]	325 ± 137 [99–861]
	BQ	12	12.9 ± 4.5 [6.9–20.4]	346 ± 174 [186–720]
	Acc	211	13.5 ± 5 [3–28.3]	282 ± 106 [99–685]
*N*. *diadema*	Acc	19	17.1 ± 6.5 [10.3–35.4]	343 ± 98 [158–544]
	Chase_cs	80	20.5 ± 6 [8.7–39.5]	323 ± 51 [152–521]
	Chase_hs	205	23.2 ± 6.7 [6.4–41]	332 ± 42 [169–404]
*N*. *sammara*	Acc	65	14.8 ± 4.4 [6.2–27]	292 ± 87 [117–503]
	Chase_cs	285	14.1 ± 4.1 [5.6–30.3]	278 ± 65 [76–902]
	Chase_hs	486	14.7 ± 6 [1.9–48.3]	281 ± 70 [52–826]
	Cp	475	14.7 ± 5.7 [3.6–48.7]	291 ± 73 [70–779]
	BC	36	15.4 ± 5.3 [8.4–30.6]	286 ± 79 [120–421]
*S*. *seychellense*	Acc	51	15.3 ± 5.4 [3.7–34]	331 ± 150 [76–791]
	Chase_cs	100	15.4 ± 4.9 [5.4–27.2]	309 ± 111 [210–714]
	Chase_hs	63	13.7 ± 3.2 [7.3–21.5]	286 ± 101 [58–756]
	BC	32	14.6 ± 5.2 [4.6–22.9]	354 ± 147 [222–867]
*S*. *spiniferum*	Acc	26	21.5 ± 10.9 [10.1–54.1]	229 ± 133 [29–667]
	Chase_cs	36	25.3 ± 8 [10.5–46]	169 ± 83 [64–451]
	Chase_hs	256	21 ± 9.6 [7.7–67.2]	191 ± 95 [76–750]
	BC	23	21.7 ± 9.5 [8.6–43.4]	277 ± 90 [35–386]

n = number of sounds.

**Table 5:** Summary of mean ± sd and [min–max values], calculated for each behaviour and species, for the different acoustical variables of sounds T2 (n = 252).

Species	Behaviour	n	Sound duration (ms)	Fundamental frequency (Hz)	Dominant frequency (Hz)	Duration of the final pulse (ms)	Period (ms)
*M*. *kuntee*	Acc	17	15.7 ± 5.7 [8.6–32.3]	-	331 ± 212 [169–884]	8.4 ± 3.2 [4.1–17.2]	7.4 ± 2.8 [4.4–15.4]
	Chase_cs	49	12.6 ± 3.9 [7.5–26.2]	-	327 ± 156 [158–867]	7.2 ± 2.5 [3.8–14.2]	5.4 ± 2.3 [2.3–15.9]
	Chase_hs	11	11.4 ± 2.3 [6.8–15]	-	469 ± 217 [152–796]	6.2 ± 1.4 [3.4–8.3]	5.3 ± 1.8 [3.2–9]
	BC	14	14 ± 5.4 [6.5–25.2]	-	403 ± 159 [181–650]	7 ± 2.9 [3.1–12.9]	7 ± 3 [3.4–12.5]
*M*. *violacea*	Acc	14	21.9 ± 9.7 [6.6–38]	-	335 ± 185 [111–761]	12.8 ± 6.6 [3.3–25]	9.2 ± 4 [3.3–17.5]
	Chase_cs	54	20.3 ± 5.7 [8–32.6]	115 [115–115]	230 ± 123 [82–650]	11 ± 4.1 [3.3–21.8]	9.3 ± 2.8 [4.1–14.1]
	Chase_hs	23	24.9 ± 11.3 [9.7–45.2]	-	194 ± 65 [93–369]	13.6 ± 6.7 [4.8–30.3]	11.5 ± 6.1 [4.2–21.4]
*N*. *diadema*	Acc	9	36.2 ± 7.3 [26.1–46.6]	96 ± 63 [41–187]	179 ± 147 [29–386]	19.9 ± 5.2 [12–27.1]	16.3 ± 3.1 [13–21.8]
*N*. *sammara*	Chase_cs	5	26.2 ± 11.7 [10.6–38]	138 ± 111 [59–216]	202 ± 84 [65–292]	11.5 ± 4.1 [6.1–17]	14.7 ± 7.7 [4.7–22.2]
	Chase_hs	12	29.1 ± 13.7 [8.7–53.9]	-	278 ± 207 [113–890]	16 ± 8.8 [4–36.6]	13.2 ± 6.9 [4.8–28.5]
	Cp	9	52.6 ± 78.3 [17.5–260.7]	188 [188–188]	274 ± 65 [193–410]	13.8 ± 3.9 [8.6–21.2]	13.6 ± 3.3 [8.8–18.3]
*S*. *spiniferum*	Acc	14	28.2 ± 16.4 [12.5–70.8]	116 ± 54 [41–175]	225 ± 200 [41–685]	15.9 ± 10.2 [6–46.4]	12.3 ± 7.4 [5.1–28.7]
	Chase_hs	16	25.4 ± 12.4 [13.6–51.7]	135 ± 34 [65–181]	213 ± 149 [111–708]	13.9 ± 9 [5.8–38.1]	14.6 ± 15.1 [4.7–68.3]
	BC	5	30.8 ± 10.6 [16.4–43.1]	-	282 ± 131 [52–369]	19 ± 8.2 [10–28.6]	11.9 ± 3.1 [6.5–14.6]

n = number of sounds.

**Table 6:** Summary of mean ± sd and [min–max values], calculated for each behaviour and species, for the different acoustical variables of sounds T3a (n = 10).

Species	Behaviour	n	Sound duration (ms)	Fundamental frequency (Hz)	Dominant frequency (Hz)	Duration of the final pulse (ms)	Period (ms)	Number of pulses
*M*. *violacea*	Acc	2	168.8 ± 10.5 [161.4–176.2]	90 ± 43 [59–120]	178 ± 83 [120–237]	20 ± 6.7 [15.3–24.8]	10.3 ± 1.8 [9.1–11.6]	15.5 ± 2.1 [14–17]
	Chase_cs	1	350.2 [350.2–350.2]	31 [31–31]	190 [190–190]	46.3 [46.3–46.3]	33.8 [33.8–33.8]	10 [10–10]
*N*. *diadema*	Chase_hs	2	620.8 ± 419.7 [324–917.6]	-	328 ± 11 [320–336]	42.3 ± 16 [31–53.6]	30.8 ± 12.2 [22.2–39.4]	24 ± 22.6 [8–40]
*N*. *sammara*	Chase_cs	1	230.6 [230.6–230.6]	-	205 [205–205]	11.3 [11.3–11.3]	13.7 [13.7–13.7]	17 [17–17]
	Chase_hs	1	375 [375–375]	-	240 [240–240]	13.7 [13.7–13.7]	6.6 [6.6–6.6]	56 [56–56]
	Cp	2	269.4 ± 168.5 [150.2–388.5]	-	131 ± 105 [57–205]	23.4 ± 6.7 [18.7–28.2]	23.2 ± 9.6 [16.4–30]	11 ± 2.8 [9–13]
*S*. *spiniferum*	Chase_hs	1	1501 [1501–1501]	-	160 [160–160]	9.2 [9.2–9.2]	25.5 [25.5–25.5]	60 [60–60]

n = number of sounds.

**Table 7:** Summary of mean ± sd and [min–max values], calculated for each behaviour and species, for the different acoustical variables of sounds T3b (n = 166).

Species	Behaviour	n	Sound duration (ms)	Number of pulses	Fundamental frequency (Hz)	Dominant frequency (Hz)	Duration of the final pulse (ms)	Period (ms)
*M*. *kuntee*	Acc	8	28.7 ± 15.9 [15–60.2]	5.1 ± 2 [3–9]	215 ± 59 [140–287]	271 ± 79 [187–445]	6 ± 2 [3.6–9.2]	5.2 ± 1.4 [3.5–7]
	Chase_cs	37	20.2 ± 15.4 [9–97.5]	4 ± 2.8 [3–18]	249 ± 61 [164–384]	342 ± 167 [164–785]	5.9 ± 2.3 [3.3–11.6]	4.7 ± 1.4 [2.9–7.8]
	Chase_hs	7	17.8 ± 5.2 [11.2–24.7]	3.6 ± 0.8 [3–5]	178 [178–178]	287 ± 127 [164–533]	4.7 ± 1.6 [2.6–7.5]	5 ± 0.8 [4–6.2]
*M*. *violacea*	Acc	32	48.1 ± 22.7 [11–117]	6.6 ± 3.1 [3–18]	172 ± 37 [71–234]	223 ± 85 [128–445]	9.1 ± 2.9 [4–13.8]	6.9 ± 1.4 [3.5–9.3]
	Chase_cs	45	36.3 ± 24.9 [13.5–121.8]	4.9 ± 3.5 [3–19]	166 ± 50 [66–269]	205 ± 66 [117–379]	10.3 ± 3.6 [5–18]	6.6 ± 1.4 [3.8–11.3]
	Chase_hs	13	39.8 ± 23.1 [18.5–104.2]	5.8 ± 3.3 [3–14]	164 ± 40 [84–228]	237 ± 86 [128–440]	9.7 ± 2.1 [6.1–13.3]	6.2 ± 1.1 [4.4–7.6]
*N*. *sammara*	Chase_cs	5	38.9 ± 24.7 [17.9–78.9]	4.4 ± 2.2 [3–8]	199 [199–199]	191 ± 31 [164–240]	11 ± 4.2 [6.9–17.7]	7.2 ± 2.2 [5.4–10.8]
	Chase_hs	10	33.5 ± 25.3 [17–103.8]	5.5 ± 3.9 [3–16]	172 ± 21 [152–199]	258 ± 70 [176–375]	7.8 ± 2.8 [4.5–12.6]	5.5 ± 0.7 [4.4–6.5]
*S*. *spiniferum*	Chase_hs	9	47.4 ± 25.9 [14.6–88.4]	6.9 ± 3.4 [3–12]	156 ± 57 [36–205]	188 ± 57 [146–328]	10.6 ± 7.4 [4.4–29.3]	6 ± 1.2 [4.2–7.7]

n = number of sounds.

**Table 8:** Summary of mean ± sd and [min–max values], calculated for each behaviour and species, for the different acoustical variables of sounds T3c (n = 78).

Species	Behaviour	n	Sound duration (ms)	Number of pulses	Fundamental frequency (Hz)	Dominant frequency (Hz)	Duration of the final pulse (ms)	Period (ms)
*M*. *violacea*	Acc	21	44.6 ± 11.8 [26.8–84.2]	3.8 ± 1 [3–6]	182 ± 55 [76–222]	201 ± 35 [103–234]	15.8 ± 6.8 [6.5–29.3]	10.7 ± 2.8 [7.5–17.7]
	Chase_cs	17	43.4 ± 18.9 [23–89.3]	4.5 ± 2.1 [3–10]	117 ± 28 [87–171]	173 ± 63 [87–300]	11.2 ± 3.5 [6.7–18.7]	9.3 ± 1.1 [7.4–11.3]
	Chase_hs	6	50.4 ± 16.4 [32.2–71.2]	5.2 ± 1.6 [4–8]	114 ± 27 [87–140]	236 ± 125 [111–462]	8 ± 1 [6.7–9.6]	10.2 ± 2.6 [8.1–14.9]
*N*. *sammara*	Acc	8	39.1 ± 15.5 [18.2–57.5]	3.9 ± 1.1 [3–6]	107 ± 17 [87–118]	196 ± 91 [65–339]	10.9 ± 4.8 [6.1–20.7]	9.9 ± 3.2 [6.2–15.9]
	Chase_hs	13	58.6 ± 28.1 [28.6–132.5]	6.2 ± 4.1 [3–18]	122 ± 37 [58–169]	266 ± 137 [64–521]	12.1 ± 4.8 [7.5–26]	10.2 ± 3.7 [6.2–19.6]
*S*. *spiniferum*	Chase_cs	6	60.6 ± 28.3 [37–115.6]	3.2 ± 0.4 [3–4]	60 ± 10 [46–70]	147 ± 65 [58–199]	17.9 ± 5.4 [11.3–25]	19.4 ± 5.6 [15.3–30.2]
	Chase_hs	7	58.8 ± 12.4 [47.5–81.9]	3 ± 0 [3–3]	76 ± 14 [59–97]	238 ± 104 [134–421]	26.4 ± 7.6 [18.9–41.8]	15.7 ± 2.9 [12.2–20.4]

n = number of sounds.

### Comparison between T3c and HH sounds

Among all the naturally occurring sounds recorded in the field, the sounds T3c could potentially correspond to the type of sound produced by HH fish. Therefore, comparisons of these HH sounds were made with respect to the T3c sound category.

**Fig 5:**
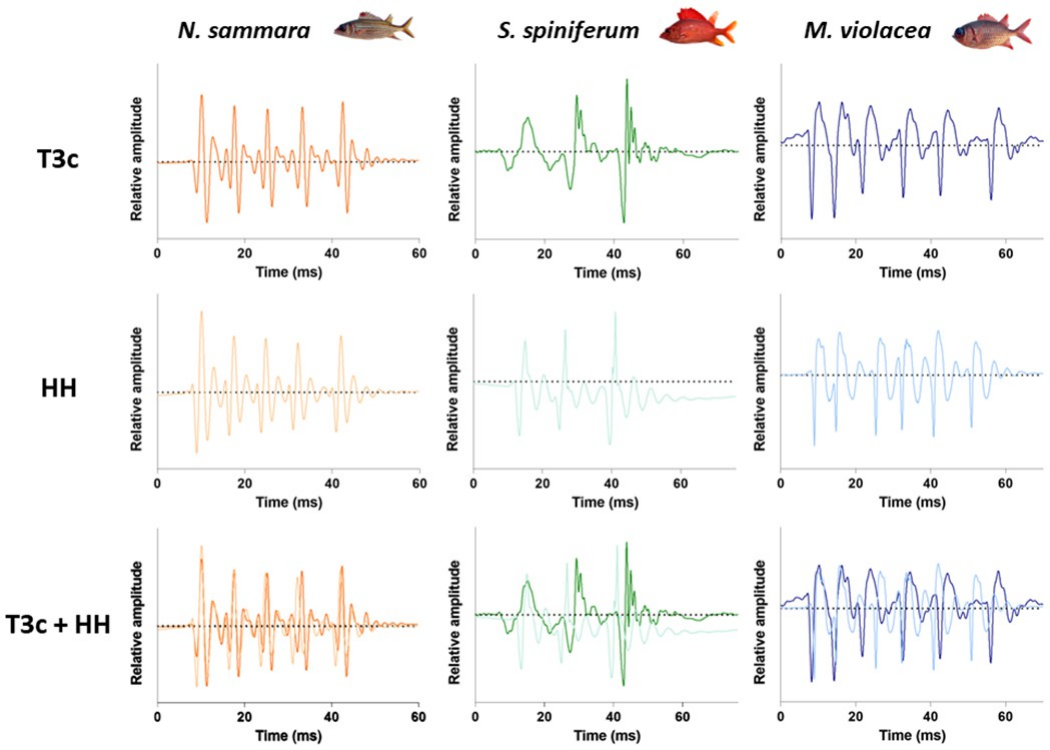
Oscillograms of T3c and HH sounds produced by *N*. *sammara*, *S*. *spiniferum* and *M*. *violacea*, and their superposition.

**Fig 6:**
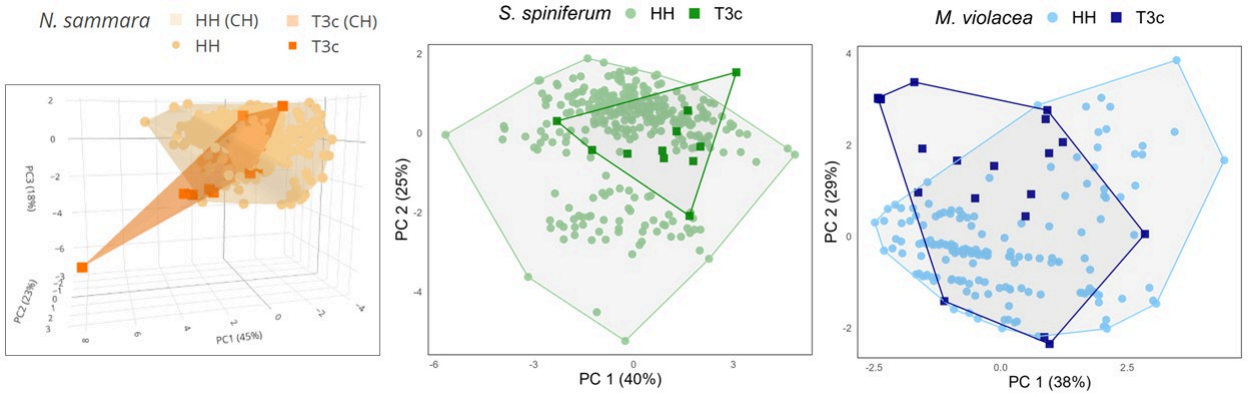
Scatterplots of the first two or three principal components (PC1, PC2, PC3) performed with the 6 acoustical variables of HH and T3c sounds in *N*. *sammara*, *S*. *spiniferum* and *M*. *violacea*. CH = convex hull. Interactive 3D scatterplot is available in S1 Data.

The comparison of the oscillograms of T3c sounds and HH sounds shows that these sounds are built in the same way (Fig 5). However, a complete correspondence could not be established for any of the three species investigated, most probably because the behavioural context was not essentially the same. In *N*. *sammara* and *M*. *violacea*, univariate statistical analyses indicate that, although being in the same range, T3c and HH sounds differ in several acoustical variables (Mann-Whitney-Wilcoxon tests, *p* < 0.05; Table 9; S25 and S26 Tables). In *S*. *spiniferum*, differences were only found in terms of fundamental and dominant frequencies (Mann-Whitney-Wilcoxon tests, *p* < 0.05; Table 9; S25 and S26 Tables), while sound duration, number of pulses, period and duration of the last pulse did not diverge. For *N*. *sammara*, the first three principal components (PC) of the PCA performed on HH and T3c sounds accounted for 45, 23 and 18% of the variability, for a cumulative explained amount of variation of 86%. For *S*. *spiniferum* and *M*. *violacea*, the two first PCs accounted for 40 and 25% and 38 and 29%, respectively. For all species, the sound duration, fundamental frequency, pulse period and duration of the last pulse mostly contributed to PC1. The number of pulses and the dominant frequency were principally associated with PC2 for *M*. *violacea* and *S*. *spiniferum*, whereas they were mainly associated with PC2 and PC1, respectively, for *N*. *sammara*. Variable correlation plots are available in S1 Fig. For all three species, there is a clear overlap between HH and T3c sounds (Fig 6).

**Table 9:** Summary of mean ± sd and [min–max values], calculated for the different acoustical variables of both sounds T3c and sounds produced by hand-held (HH) fish in standardized conditions at sea, for each species.

Species	Sound type	n	Sound duration (ms)	Number of pulses	Fundamental frequency (Hz)	Dominant frequency (Hz)	Duration of the final pulse (ms)	Period (ms)
*M*. *violacea*	HH	181	63.4 ± 14 [29.9–105.5]	6.8 ± 1.5 [3–9]	118 ± 14 [79–156]	241 ± 67 [102–398]	11.7 ± 6.8 [4–28.8]	9.1 ± 2.2 [6.7–18.3]
	T3c	44	44.9 ± 15.3 [23–89]	4.2 ± 1.6 [3–10]	149 ± 54 [76–222]	195 ± 66 [87–462]	12.9 ± 5.9 [6.5–29.3]	10.1 ± 2.3 [7.4–17.7]
*N*. *sammara*	HH	449	50.6 ± 14.6 [27.1–125.2]	5.4 ± 1.4 [3–16]	133 ± 16 [62–187]	384 ± 148 [209–922]	15.3 ± 5.8 [3.4–34]	7.6 ± 0.9 [5.6–11.7]
	T3c	21	51 ± 25.5 [18–132]	5.3 ± 3.5 [3–18]	118 ± 33 [58–169]	240 ± 124 [64–521]	11.6 ± 4.7 [6.1–26]	10.1 ± 3.4 [6.2–19.6]
*S*. *spiniferum*	HH	323	61.7 ± 10.2 [35.6–104.7]	3.2 ± 0.4 [3–5]	55 ± 12 [24–113]	241 ± 40 [102–333]	24.9 ± 5.5 [11.3–40.2]	16.3 ± 3.4 [8.3–33.1]
	T3c	13	59.6 ± 20.4 [37–116]	3.1 ± 0.3 [3–4]	70 ± 15 [46–97]	196 ± 97 [58–421]	22.5 ± 7.7 [11.3–41.8]	17.4 ± 4.6 [12.2–30.2]

n = number of sounds.

## Discussion

Understanding the messages conveyed by teleost fish in their natural environment and determining if specific sounds are associated with distinct behaviours remain largely underexplored. One notable contribution of this research is the documentation of acoustical events associated with the execution of specific behaviours. Usually, a fish emits a consistent type of sound during a particular behaviour, which, depending on motivation, may be repeated multiple times over a defined period. However, in some cases, an acceleration in the pulse production can lead to variations in sound during the same behaviour. For example, in Gobiidae, tonal or complex sounds are produced by an acceleration in the emission of drumming [11]. In different holocentrid species, sounds that are produced during an acoustic event, can range from a solitary sound to a complex arrangement of sounds, either similar or varied in types supporting the lack of direct relationships between a behaviour and a kind of sound. In other words, the behaviour cannot be inferred from the sound. To the best of our knowledge, this observation has not been previously described in vocal teleosts. Different studies have reported that fish can produce typical kinds of sound in association to particular behaviours [9–11]. Here we show that the situation is different among the Holocentridae. Regardless of the species, events associated with different behaviours are not stereotyped and can be composed of various sound types, the distribution of which appearing to be random.

### Behaviours associated with sound production

In the literature, holocentrids have been reported to produce sounds in several social contexts such as territory defence, chasing and escape behaviours observed during fish introduction experiments in tanks but also during alarm/vigilance behaviours [17, 22–24, 28]. Responses of holocentrids included dashing at the intruder, fleeing, mobbing towards a predator (i.e., moray eel) and lateral displays. We have observed the same behaviours in the wild, but we have also identified a different competition behaviour, characterized by parallel swimming in two conspecifics. In this behaviour, the pair of fish could swim straight or in circle, corresponding to the lateral display and circling behaviours described in *H*. *rufus* and *M*. *violacea*, respectively [22, 23]. However, our data indicate that Holocentridae can use sounds in behaviours beyond those observed during agonistic interactions since sounds were also produced during three types of social signalling behaviours: broadcasting, acceleration and body quivering. Moreover, while more occurrences of conspecific chase were recorded for species of the subfamily Myripristinae, more heterospecific chases were observed for species of the subfamily Holocentrinae. This differentiation in behaviours could be explained by the ecology of the species among the two subfamilies. Indeed, while some species of Holocentrinae (e.g., *H*. *rufus*) seem to be solitary and territorial, species of the Myripristinae, such as *M*. *berndti*, live mainly in non-territorial schools that can consist of several dozens of individuals depending on available space [23, 25, pers. obs.]. From many personal observations in the field, all *Myripristis* species are generally found in schools while *N*. *diadema*, *N*. *sammara*, *S*. *seychellense* and *S*. *spiniferum* are mostly solitary, although a few individuals can be observed inhabiting the same shelter. Horch and Salmon [23] discussed differences in sound production between territorial and non-territorial holocentrid species. Sound production would help territorial individuals to maintain territories and promote the survival of all fish in adjacent areas. It would also help to maintain distances between individuals living in aggregations which could be beneficial for two reasons: (1) to increase the detection of a predator and (2) to decrease the risk of multiple individuals being captured during predator attacks. Although new behaviours associated with sound production have been described for the first time in this study, information regarding acoustic communication in reproduction contexts are still lacking. Since acoustic communication has been shown to be significant in this taxa, reproductive behaviours are most likely associated with sound production. The absence of observed reproductive behaviour during daylight hours suggests that holocentrids likely reproduce at night in open water [30].

In many teleosts, such as members of the Pomacentridae [9, 10, 31], gobioids [31, 32], Cottidae [33], and Cichlidae [34], sounds can be stereotyped to particular behaviours. In our study, such relationships between sound types and behaviours were not found in holocentrids. Indeed, all sound types (T1, T2, T3a, T3b and T3c) were produced both during agonistic and social signalling behaviours in all species, and acoustical events were often composed of several sound types without any phraseology or stereotyped structure. This suggests that sounds would not indicate precise behaviours in holocentrids but would rather serve to enhance visual communication, at least during the day. In *H*. *rufus*, different behaviours, such as nips, shudders, head shakes, chases, lateral display and fin erection occurred as single elements with or without grunts [22], a finding that supports this hypothesis. The use of sound to reinforce behaviours was also reported in the Nile tilapia, *O*. *niloticus* [12]. During symbiotic interactions of several holocentrid species with cleaner fishes of the genus *Labroides*, a lack of stereotypy in the sounds had already been observed. To end or refuse the association, holocentrids emitted different acoustical signals that additionally lacked a distinct structure [26]. This absence of code could be explained by the heterospecific nature of the communication between holocentrids and the *Labroides*. Interestingly, the use of a particular call in different behavioural contexts (e.g., confrontation with predators, interactions with mates and territorial rivals, aggregation in foraging flocks) has also been showed in songbirds [35–37]. While the antipredator function can be rather obvious, the function of such signals in nonpredator contexts is sometimes unclear. However, the repetition rate of calls could be a cue to differentiate between contexts, with rapidly repeated calls in situations involving widely threatening predators and slower rate in other social interactions [37]. Moreover, the association of visual and acoustic behaviours may contribute to the differentiation of the conveyed signal. In the cichlid *Metriaclima zebra*, combining a sound with a visual behaviour results in a lower level of aggression compared to exposure to isolated visual signals. This suggests that acoustic signals used during a dispute may complement visual displays to modulate males’ behaviour, thereby reducing their aggressiveness and the risk of escalated conflicts [38]. Besides the context of sound emission, the complementarity of different signal types is evidenced in acoustic fishes [39]. Acoustic communication is part of a complex system that allows conspecific and heterospecific individuals to communicate together in a multimodal way (acoustically, visually, chemically, etc.). It is the combination of the different modalities that most likely enable fishes to communicate efficiently.

In contrasts with the lack of stereotypy we have described, previous studies have showed that HH sounds produced by holocentrids [15, 21] were all of a single type. These examples show that sounds produced within the same environmental constraints, which is not the case in our recordings in the wild, can be similar.

### Comparison between T3c and HH sounds

Different acoustic features as well as the oscillogram traces (Fig 5) support that HH sounds correspond to T3c sounds recorded in the field despite some differences in the statistical analyses. Recording and environmental conditions can themselves easily explain most of the statistical differences found within acoustical features. Sounds produced by HH fish were recorded in a quiet environment, in the same behavioural context, directed at the same receiver and at the same distance and relative position of the fish with respect to the hydrophone. In field recordings, the behavioural contexts and, as a result, the motivations for producing sounds differed, in addition to the sounds being targeted at different potential receivers and the fish not maintaining a consistent distance and orientation relative to the hydrophone. It is also interesting to note that in the wild, T3c sounds are produced during several kinds of behaviours, while this is the only sound type that fish produce when they are hand-held. It also highlights that the technique of holding fish by hand, used in various studies, has the advantage of limiting variability, thus facilitating comparison between species.

### Reconciling the terminology of sound types in holocentrids

The descriptions of the different sound types produced by holocentrids date more than 5 decades [17, 22–24] but often lacked comprehensiveness and quantitative information to enable their comparisons between studies, including ours. Five sound types were described in holocentrids: thump, grunt, staccato, growl and knock.

If the absence of distinct organization in the acoustical events, often composed of several sound types, may at first be surprising, it reveals an important aspect of sound production: for all sound types, pulses seem to be produced similarly because of the contraction of sonic muscles. Sounds T1, T2, and T3 would be produced by the same mechanism but with variation in the motor pattern, thus modifying the frequency rate of pulses. Sound type T3 could therefore simply be a repetition of sound type T1, with a frequency that categorizes several sound types. This new perspective regarding sound types would indicate that the various onomatopoeias provided by the different authors to represent different sound types would primarily rely upon the motivation of the emitter that would modulate the frequency at which the pulses are emitted. It could also explain that different sound types have been described for the same behaviour or that different behaviours have the same sound types (Table 1). A parallel can be drawn here with different types of sounds emitted by some Gobiidae [11] and Pomacentridae [40], where multi-pulsed sound types result from iterations of isolated pulses.

**Fig 7:**
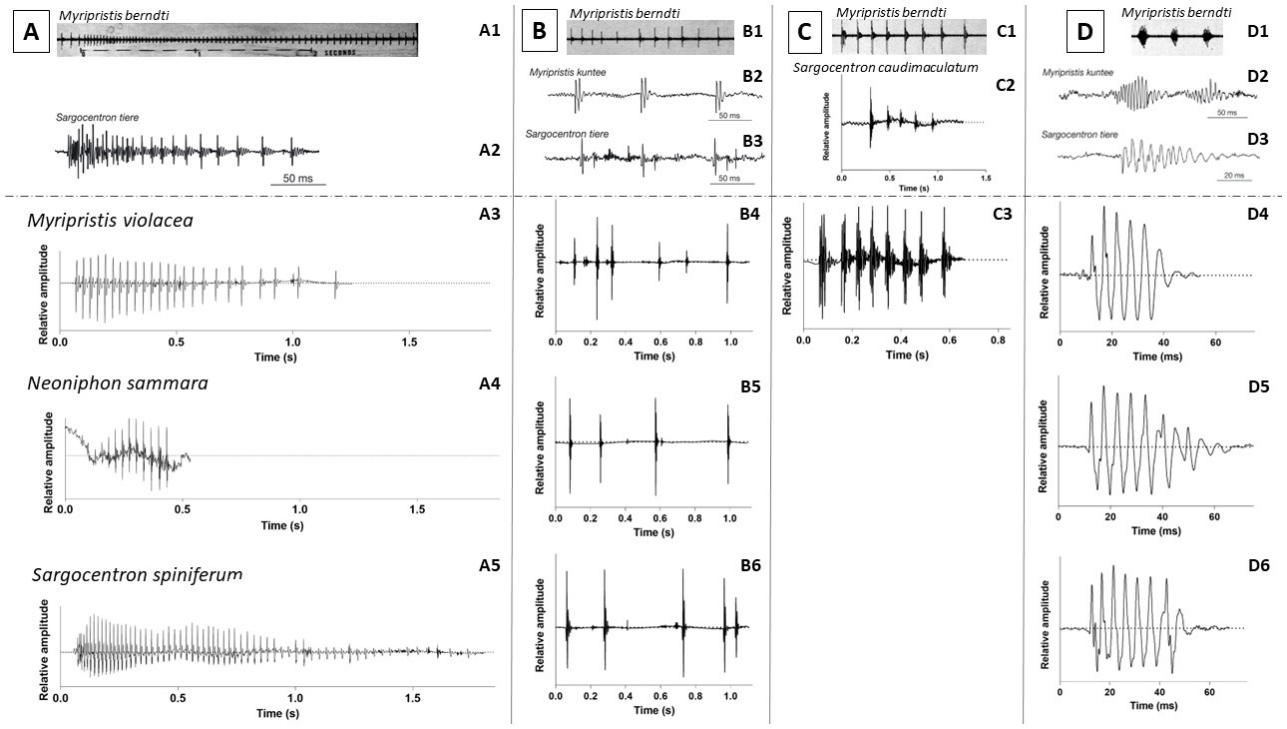
Oscillograms of (A) growl, (B) knock, (C) staccato and (D) grunt sounds of several species of Holocentridae. (Upper part) From previous studies (Salmon, 1967; Tricas and Boyle, 2014; Banse *et al*., 2024a), for *M*. *berndti* (A1, B1, C1, D1), *M*. *kuntee* (B2, D2), *S*. *tiere* (A2, B3, D3) and *S*. *caudimaculatum* (C2). (Down part) From this study, for *M*. *violacea* (A3, B4, C3, D4), *N*. *sammara* (A4, B5, D5) and *S*. *spiniferum* (A5, B6, D6).

To clarify the different terms used in the literature regarding the different sound types produced by holocentrids, we established relationships between our sound types and the onomatopoeias (Fig 7):

1. Knocks, or short duration sounds produced at irregular intervals, would correspond to sounds T1 and T2 (Fig 7B). In agreement with previous studies [24, 28], knocks were the predominant sound type produced in this study for all behaviours and species. Their mean durations range between 6 and 34 ms for T1 sounds and between 11 and 52 ms for T2 sounds.
2. Growls would correspond to T3a sounds (Fig 7A). They have a long duration (> 150 ms) that can reach up to almost 2 seconds and are characterized by a decrease in pulse rate towards the end of the call. These sounds are rarely produced.
3. Grunts are sounds of a mean duration that can vary between 15 and 85 ms, depending on the number of pulses (3 to 19) (Fig 7D). They usually show harmonics. Grunts would correspond to T3b sounds.
4. Staccatos were first described as a sound consisting of a variable number of grunts repeated rapidly [22] but no visual nor quantitative data were provided by the authors. An oscillogram of this sound type was provided a few years later (Fig 7C1) [24]. Recently, Banse *et al*. [25] reported the production of staccatos by *S*. *caudimaculatum* at night with increasing calling rate during acoustic mobbing behaviour performed against a moray-eel (Fig 7C2). We recorded, although very unfrequently, the so-called staccatos made of several grunts (Fig 7C3).
5. Thumps are sounds of a mean duration that can vary between 18 and 133 ms, depending on the number of pulses (3 to 18) that correspond to T3c sounds (Fig 5). Similarly to the grunts, thumps usually show harmonics. Their pulses are however more discernible than in grunts.

## Conclusions

The literature on sound production behaviours in fish within their natural habitats is quite sparse and it seems that there is no well-defined pattern, likely because communication has a multimodal aspect. Our study shows that all holocentrids could produce sounds in 6 behavioural contexts of both agonistic (conspecific and heterospecific chases, competition) and social signalling types (acceleration, broadcasting, body quivering), in addition to mobbing [25] and symbiotic interactions with cleaner wrasses [26]. In this family, a behaviour is not necessarily linked to a specific type of sound. Behaviours were accompanied by single pulse sounds or unusual suites of sounds that could be of the same type or different types, besides being arranged randomly. We could relate sound types to previous onomatopoeia used in the literature that lacked descriptive physical and quantitative data: knocks (T1, T2), growls (T3a), grunts (T3b), staccatos (series of T1/T2 or of T3b), and thumps (T3c). In the Holocentridae, the absence of stereotypy suggests that sounds are primarily produced to reinforce visual communication, at least during daylight behaviours. Moreover, we hypothesized that sounds of type T3c produced by free-swimming individuals correspond to those produced by HH specimens.

## Supporting information

S1 Text Detailed descriptions of the statistical comparisons of acoustical features describing the events between the different behaviours for each species. (DOCX)

S2 Text Detailed descriptions of the statistical comparisons of acoustical features describing sounds between the different behaviours, for each species. (DOCX)

S1 Data Interactive 3D scatterplot of the first three principal components (PC1, PC2, PC3) performed with the 6 acoustical variables of HH and T3c sounds in *N*. *sammara*. CH = convex hull.
(HTML)

S1 Fig Variable correlation plots for principal component analyses on HH and T3c sounds of (A) *N*. *sammara*, (B) *S*. *spiniferum* and (C) *M*. *violacea*. (TIF)

S1 Movie This sequence captures *Neoniphon diadema* performing an acceleration behaviour. (MP4)

S2 Movie This sequence captures *Neoniphon diadema* chasing a conspecific. (MP4)

S3 Movie This sequence captures *Neoniphon diadema* chasing an heterospecific (*Pempheris sp*.). (MP4)

S4 Movie This sequence captures two individuals of *Neoniphon sammara* performing a competition behaviour. (MP4)

S5 Movie This sequence captures *Myripristis berndti* performing a broadcast signal (dorsal fin erection). (MP4)

S6 Movie This sequence captures *Neoniphon microstoma* doing a body quivering. (MP4)

S1 Table Information on data collection. (DOCX)

S2 Table Numbers of acoustical events (N; above) and sounds (n; below) observed for each behaviour and species. Acc = acceleration, Chase_cs = conspecific chase, Chase_hs = heterospecific chase, Cp = competition, BC = broadcasting, BQ = body quivering. Underlined species correspond to those selected to perform the statistical analyses. For each species, behaviours whose the number acoustical events was < 5 were excluded from the analyses. Two additional events were removed from the Cp behaviour in *N*. *sammara* since they were extremely long with respect to the others.
(DOCX)

S3 Table Number (above) and frequency (below) of each sound type composing the acoustical events of each behaviour for each species. For each species, sound types whose the number of observations was < 5 were excluded from the statistical comparisons between behaviours. n = number total of sounds.
(DOCX)

S4 Table Results of Kruskal-Wallis tests on the acoustical variables of events between the different behaviours for each species. Significance level = 0.05. NS = non-significant. *P* values in bold are significant. DuE = event duration, Nsounds = number of sounds, rhyt = rhythm.
(DOCX)

S5 Table Results of the post-hoc Dunn tests on event duration (DuE) and number of sounds (Nsounds) significantly different between behaviours for each species based on Kruskal-Wallis tests. Significance level = α = 0.05. Significance threshold of the Dunn test (dunn.test function with parameter ‘altp’ = FALSE) = α/2 = 0.025. NS = non-significant. *P* values in bold are significant.
(DOCX)

S6 Table Results of ANOVA tests on event duration (DuE) and rhythm (Rhyt) in *S*. *spiniferum*. Significance level = 0.05. *P* values in bold are significant.
(DOCX)

S7 Table Results of the post-hoc Tukey’s tests on event duration (DuE) and rhythm (Rhyt) in *S*. *spiniferum*, significantly different, based on ANOVA tests, between behaviours. Significance level = 0.05. NS = non-significant. *P* values in bold are significant.
(DOCX)

S8 Table Number of unique combinations of sound types composing the acoustical events for each behaviour and species, with respect to the total number of events for each category (between brackets). (DOCX)

S9 Table Number (above) and frequency (below) of events that began with a sound of type T1 for each behaviour and species. N = number of acoustic events.
(DOCX)

S10 Table Results of the Kruskal-Wallis tests on the acoustical variables of sounds T1 between the different behaviours for each species. Significance level = 0.05. NS = non-significant. *P* values in bold are significant. Du = sound duration, fpeak = dominant frequency.
(DOCX)

S11 Table Results of the post-hoc Dunn tests on the acoustical variables of sounds T1 significantly different between behaviours for each species, based on Kruskal-Wallis tests. Significance level = α = 0.05. Significance threshold of the Dunn test (dunn.test function with parameter ‘altp’ = FALSE) = α/2 = 0.025. NS = non-significant. *P* values in bold are significant. Du = sound duration, fpeak = dominant frequency.
(DOCX)

S12 Table Results of ANOVA tests on several acoustical variables of sounds T2 between the different behaviours for each species. Significance level = 0.05. NS = non-significant. *P* values in bold are significant. Du = sound duration, lastpu = duration of the last pulse, duper = pulse period, F0 = fundamental frequency.
(DOCX)

S13 Table Results of the post-hoc Tukey’s tests on pulse period (duper) of sounds T2 in *M*. *kuntee*, significantly different between behaviours, based on ANOVA test. Significance level = 0.05. NS = non-significant. *P* values in bold are significant.
(DOCX)

S14 Table Results of the Kruskal-Wallis tests on several acoustical variables of sounds T3b between the different behaviours for each species. Significance level = 0.05. NS = non-significant. *P* values in bold are significant. Du = sound duration, npulses = number of pulses in sounds, F0 = fundamental frequency, lastpu = duration of the last pulse, fpeak = dominant frequency.
(DOCX)

S15 Table Results of the post-hoc Dunn tests on the acoustical variables of sounds T3b significantly different between behaviours for each species, based on Kruskal-Wallis tests. Significance level = α = 0.05. Significance threshold of the Dunn test (dunn.test function with parameter ‘altp’ = FALSE) = α/2 = 0.025. NS = non-significant. *P* values in bold are significant. Npulses = number of pulses in sounds, du = sound duration.
(DOCX)

S16 Table Results of ANOVA tests on several acoustical variables of sounds T3b between the different behaviours for each species. Significance level = 0.05. NS = non-significant. Duper = pulse period, F0 = fundamental frequency.
(DOCX)

S17 Table Results of the Kruskal-Wallis tests on several acoustical variables of sounds T2 between the different behaviours for each species. Significance level = 0.05. NS = non-significant. *P* values in bold are significant. Fpeak = dominant frequency, lastpu = duration of the last pulse.
(DOCX)

S18 Table Results of the post-hoc Dunn tests on the dominant frequency (Fpeak) of sounds T2 in *M*. *violacea*, significantly different between behaviours, based on Kruskal-Wallis tests. Significance level = α = 0.05. Significance threshold of the Dunn test (dunn.test function with parameter ‘altp’ = FALSE) = α/2 = 0.025. NS = non-significant. *P* values in bold are significant.
(DOCX)

S19 Table Results of the Kruskal-Wallis tests on the acoustical variables of sounds T3c between behaviours of *M*. *violacea*. Significance level = 0.05. NS = non-significant. *P* values in bold are significant. Du = sound duration, npulses = number of pulses in sounds, lastpu = duration of the last pulse, F0 = fundamental frequency, fpeak = dominant frequency, duper = pulse period.
(DOCX)

S20 Table Results of the post-hoc Dunn tests on the acoustical variables of sounds T3c significantly different between behaviours in *M*. *violacea* based on Kruskal-Wallis tests. Significance level = α = 0.05. Significance threshold of the Dunn test (dunn.test function with parameter ‘altp’ = FALSE) = α/2 = 0.025. NS = non-significant. *P* values in bold are significant. F0 = fundamental frequency, lastpu = duration of the last pulse.
(DOCX)

S21 Table Results of the T-tests performed on several acoustical variables of sounds T3c between the different behaviours of *N*. *sammara* and *S*. *spiniferum*. Significance level = 0.05. NS = non-significant. *P* values in bold are significant. Du = sound duration, lastpu = duration of the last pulse, F0 = fundamental frequency, fpeak = dominant frequency, duper = pulse period.
(DOCX)

S22 Table Results of the T-tests performed on several acoustical variables of sounds T3b between the different behaviours in *N*. *sammara*. Significance level = 0.05. NS = non-significant. *P* values in bold are significant. Du = sound duration, fpeak = dominant frequency, lastpu = duration of the last pulse, duper = pulse period.
(DOCX)

S23 Table Results of the Mann-Whitney-Wilcoxon tests performed on several acoustical variables of sounds T3b between the different behaviours in *N*. *sammara*. Significance level = 0.05. NS = non-significant. Npulses = number of pulses in sounds, F0 = fundamental frequency.
(DOCX)

S24 Table Results of the Mann-Whitney-Wilcoxon tests performed on several acoustical variables of sounds T3c between the different behaviours in *N*. *sammara* and *S*. *spiniferum*. Significance level = 0.05. NS = non-significant. Npulses = number of pulses in sounds, fpeak = dominant frequency.
(DOCX)

S25 Table Results of the T-tests performed on the duration of the last pulse (lastpu) and the pulse period (duper) of T3c sounds and hand-held (HH) sounds in *S*. *spiniferum*. Significance level = 0.05. NS = non-significant.
(DOCX)

S26 Table Results of the Mann-Whitney-Wilcoxon tests performed on several acoustical variables of T3c sounds and hand-held (HH) sounds in *N*. *sammara*, *S*. *spiniferum* and *M*. *violacea*. Significance level **=** 0.05. NS **=** non-significant. *P* values in bold are significant. Du **=** sound duration, npulses **=** number of pulses in sounds, F0 **=** fundamental frequency, fpeak **=** dominant frequency, lastpu **=** duration of the last pulse, duper **=** pulse period.
(DOCX)
