# Supplementary material for: Same calls, different meanings: Acoustic communication of Holocentridae
Source: PLoS One. 2024 Nov 21;19(11):e0312191. doi: 10.1371/journal.pone.0312191 (PMC11581312; doi:10.1371/journal.pone.0312191)
Supplement: S1 Text — (DOCX) [file pone.0312191.s001.docx]

##### *Myripristis kuntee*

In *M. kuntee*, four behaviours were investigated: conspecific and heterospecific chases, acceleration and broadcasting. No difference was observed in the acoustical variables related to the events between the different behaviours in this species (Dunn tests following Kruskal-Wallis tests, *p* > 0.025; **Table 3; Tables S4,S5**).

##### *Mypristis violacea*

In *M. violacea*, five behaviours were investigated: conspecific and heterospecific chases, acceleration, broadcasting and body quivering. Acoustic events associated with conspecific and heterospecific chases lasted significantly two to three times longer and were composed of more sounds than those associated with social signalling behaviours (acceleration, broadcasting and body quivering) (Dunn tests following Kruskal-Wallis tests, *p* < 0.025; **Table 3; Tables S4,S5**), except the conspecific chase with respect to body quivering.

##### *Neoniphon diadema*

In *N. diadema,* three behaviours were investigated: conspecific and heterospecific chases, and acceleration. Acoustic events associated with conspecific and heterospecific chases lasted longer and were composed of more sounds than those associated with acceleration (Dunn tests following Kruskal-Wallis tests, *p* < 0.025; **Table 3; Tables S4,S5**).

##### *Neoniphon sammara*

In *N. sammara,* five behaviours were investigated: conspecific and heterospecific chases, competition, acceleration and broadcasting. Acoustical events associated with agonistic behaviours (competition, conspecific and heterospecific chases) lasted significantly longer and were composed of more sounds than those associated with broadcasting (Dunn tests following Kruskal-Wallis tests, *p* < 0.025; **Table 3; Tables S4,S5**). Additionally, acoustical events associated with competition lasted significantly longer and were composed of more sounds than those associated with the two other agonistic behaviours and acceleration (Dunn tests following Kruskal-Wallis tests, *p* < 0.025; **Table 3; Tables S4,S5**). Finally, the number of sounds composing the acoustical events associated with conspecific chase was higher than the number of sounds composing the acoustical events associated with acceleration, although their duration was not statistically different (Dunn tests following Kruskal-Wallis tests, *p* < 0.025; **Table 3; Tables S4,S5**).

##### *Sargocentron seychellense*

In *S. seychellense,* four behaviours were investigated: conspecific and heterospecific chases, acceleration and broadcasting. Similarly to *M. kuntee,* no difference was observed in the acoustical variables related to the events between the different behaviours in this species *(*Kruskal-Wallis tests, *p* > 0.05; **Table 3; Table S4**).

##### *Sargocentron spiniferum*

In *S. spiniferum,* four behaviours were investigated: conspecific and heterospecific chases, acceleration and broadcasting. Acoustical events associated with heterospecific chase were composed of more sounds than those associated with broadcasting (Dunn test following Kruskal-Wallis test, *p* < 0.025; **Table 3; Tables S4-S7**).
