# Supplementary material for: Same calls, different meanings: Acoustic communication of Holocentridae
Source: PLoS One. 2024 Nov 21;19(11):e0312191. doi: 10.1371/journal.pone.0312191 (PMC11581312; doi:10.1371/journal.pone.0312191)
Supplement: S2 Text — (DOCX) [file pone.0312191.s002.docx]

Statistical comparisons could not be performed between behaviours for different species because only ten sounds of type T3a were recorded altogether. A summary of the acoustical features of these sounds is, however, provided in **Table S6**.

#### Myripristis kuntee

In *M. kuntee,* sound types T1 and T2 were compared between 4 behaviours (acceleration, broadcasting, conspecific and heterospecific chases) and sound type T3b between 3 behaviours (acceleration, conspecific and heterospecific chases). Few significant differences were found: the dominant frequency of T1 sounds was lower in conspecific chase than in both broadcasting and acceleration (Dunn tests following Kruskal-Wallis tests, *p* < 0.025; **Table 4; Tables S10,S11**); the pulse period of T2 sounds was longer in acceleration than in conspecific chase (Tukey’s test following Anova, *p* < 0.025; **Table 5; Tables S12,S13**); the number of pulses in T3b sounds was larger in acceleration than in conspecific chase (Dunn tests following Kruskal-Wallis tests, *p* < 0.025; **Table 7; Tables S14,S15**), but there was no difference in the pulse period of these sounds between behaviours (Anova test, *p* > 0.05; **Table 7; Table S16**).

#### Myripristis violacea

In *M. violacea*, type T1 sounds were compared between all behaviours, except competition, and sound types T2, T3b and T3c between 3 behaviours (acceleration, conspecific and heterospecific chases). Several significant differences were observed: T1 sounds produced during conspecific chase lasted longer than those produced during heterospecific chase, acceleration and broadcasting (Dunn test following Kruskal-Wallis test, *p* < 0.025; **Table 4; Tables S10,S11**); the dominant frequency of T1 sounds was also lower in conspecific chase than in the other behaviours (Dunn test following Kruskal-Wallis test, *p* < 0.025; **Table 4; Tables S10,S11**). Significant differences were also observed in terms of dominant frequency between T1 sounds produced during acceleration, broadcasting and heterospecific chase (Dunn test following Kruskal-Wallis test, *p* < 0.025; **Table 4; Tables S10,S11**); the dominant frequency of T2 sounds was also significantly higher in acceleration than in conspecific and heterospecific chases (Dunn test following Kruskal-Wallis test, *p* < 0.025; **Table 5; Tables S17,S18**); both sound duration and the number of pulses of T3b sounds were higher in acceleration than in conspecific chase (Dunn tests following Kruskal-Wallis tests, *p* < 0.025; **Table 7; Tables S14,S15**); finally, the duration of the final pulse in sounds T3c was shorter in heterospecific chase than in acceleration and conspecific chase (Dunn tests following Kruskal-Wallis tests, *p* < 0.025; **Table 8; Tables S19,S20**).

#### Sargocentron seychellense

*Sargocentron seychellense* produced almost only sounds of type T1. Those were compared between 4 behaviours (acceleration, broadcasting, conspecific and heterospecific chases). The dominant frequency of these sounds was significantly higher during broadcasting than during both conspecific and heterospecific chases (Dunn test following Kruskal-Wallis test, *p* < 0.025; **Table 4; Tables S10,S11**).

#### Sargocentron spiniferum

In *S. spiniferum*, type T1 sounds were compared between 4 behaviours (acceleration, broadcasting, conspecific and heterospecific chases), T2 sounds between 3 behaviours (acceleration, broadcasting and heterospecific chase), and T3c sounds between conspecific and heterospecific chases. Few significant differences were observed: T1 sounds produced during conspecific chase lasted longer than those produced during acceleration and heterospecific chase (Dunn test following Kruskal-Wallis test, *p* < 0.025; **Table 4; Tables S10,S11**); the dominant frequency of T1 sounds was also higher during broadcasting than during acceleration, conspecific and heterospecific chases (Dunn test following Kruskal-Wallis test, *p* < 0.025; **Table 4; Tables S10,S11**); finally, the duration of the last pulse in T3c sounds was longer in heterospecific chase than in conspecific chase (T-test, *p* < 0.05; **Table 8; Table S21**).

#### Neoniphon diadema

In *N. diadema,* T1 sounds were compared between three behaviours (acceleration, conspecific and heretospecific chases). The statistical comparison indicates that T1 sounds produced during acceleration are significantly shorter than those produced during conspecific and heterospecific chases (Dunn tests following Kruskal-Wallis tests, *p* < 0.025; **Table 4; Tables S10,S11**).

#### Neoniphon sammara

In *N. sammara*, type T1 sounds were compared between all behaviours except body quivering, T2 and T3a sounds between three behaviours (competition, conspecific and heterospecific chases), T3b sounds between conspecific and heterospecific chases, and T3c sounds between acceleration and heterospecific chase. Only two significant differences were found among all comparisons (**Tables S10,S11,S12,S17,S21,S22,S23,S24**): the dominant frequency of T1 sounds was slightly higher during competition than during both conspecific and heterospecific chases (Dunn tests following Kruskal-Wallis tests, *p* < 0.025; **Table 4; Tables S10,S11**) and the pulse period of sounds T3b was longer in conspecific chase than in heterospecific chase (T-test, *p* < 0.05; **Table 7; Table S22**).
