# Supplementary figures and images for: Same calls, different meanings: Acoustic communication of Holocentridae
Source: PLoS One. 2024 Nov 21;19(11):e0312191. doi: 10.1371/journal.pone.0312191 (PMC11581312; doi:10.1371/journal.pone.0312191)

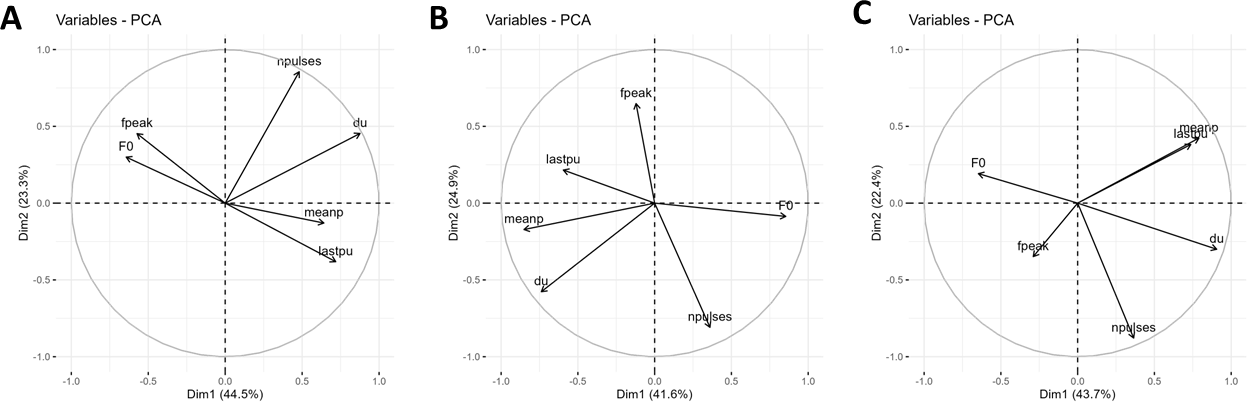

Supplement: S1 Fig — (TIF) [file pone.0312191.s004.tif]
