# Supplementary material for: Same calls, different meanings: Acoustic communication of Holocentridae
Source: PLoS One. 2024 Nov 21;19(11):e0312191. doi: 10.1371/journal.pone.0312191 (PMC11581312; doi:10.1371/journal.pone.0312191)
Supplement: S1 Table — (DOCX) [file pone.0312191.s011.docx]

| **Session** | **Region** | **Location** | **# shelter** | **Recording time (min)** | **Date** |
| --- | --- | --- | --- | --- | --- |
| 1 | Guam | Agat | 1 | 163 | 13/12/2021 |
| 2 |  | Fish Eye | 1 | 61 | 27/11/2021 |
| 3 |  | Tumon Bay | 1 | 75 | 01/12/2021 |
| 4 |  | Tumon Bay | 2 | 79 | 01/12/2021 |
| 5 |  | Tumon Bay | 3 | 79 | 04/12/2021 |
| 6 |  | Tumon Bay | 4 | 103 | 05/12/2021 |
| 7 |  | Tumon Bay | 5 | 65 | 05/12/2021 |
| 8 |  | Tumon Bay | 6 | 55 | 08/12/2021 |
| 9 |  | Tumon Bay | 7 | 96 | 08/12/2021 |
| 10 |  | Tumon Bay | 8 | 55 | 08/12/2021 |
| 11 |  | Tumon Bay | 9 | 48 | 08/12/2021 |
| 12 |  | Tumon Bay | 10 | 27 | 11/12/2021 |
| 13 |  | Tumon Bay | 13 | 50 | 11/12/2021 |
| 14 | French Polynesia | Papetoai | 1 | 89 | 19/11/2020 |
| 15 |  | Papetoai | 2 | 93 | 06/11/2020 |
| 16 |  | Papetoai | 3 | 76 | 06/11/2020 |
| 17 |  | Papetoai | 4 | 67 | 19/11/2020 |
| 18 |  | Tiahura | 1 | 70 | 25/11/2020 |
| 19 |  | Papetoai | 6 | 64 | 17/11/2020 |
| 20 |  | Piha’ena | 1 | 101 | 04/11/2020 |
| 21 |  | Temae | 1 | 74 | 24/11/2020 |
| 22 |  | Haapiti | 1 | 61 | 01/12/2020 |
| 23 |  | Haapiti | 2 | 97 | 16/11/2020 |
| 24 |  | Haapiti | 3 | 70 | 23/11/2020 |
| 25 |  | Tiahura | 2 | 88 | 24/11/2020 |
| 26 |  | Tiahura | 3 | 88 | 27/11/2020 |
| 27 |  | Piha’ena | 1 | 75 | 04/11/2020 |
| 28 |  | Cook’s Bay | 2 | 58 | 14/10/2020 |
| 29 |  | Haapiti | 4 | 80 | 01/12/2020 |
| 30 |  | Haapiti | 5 | 79 | 29/10/2020 |
| 31 |  | Papetoai | 5 | 71 | 06/11/2020 |
| 32 |  | Papetoai | 6 | 81 | 06/11/2020 |
| 33 |  | Papetoai | 7 | 62 | 17/11/2020 |
| 34 |  | Papetoai | 8 | 62 | 17/11/2020 |
| 35 |  | Papetoai | 9 | 44 | 19/11/2020 |
| 36 |  | Papetoai | 10 | 30 | 19/11/2020 |
| 37 |  | Temae | 2 | 64 | 24/11/2020 |
| 38 |  | Tiahura | 1 | 40 | 30/10/2020 |
| 39 |  | Tiahura | 4 | 70 | 24/11/2020 |
| 40 |  | Tiahura | 5 | 76 | 25/11/2020 |
| 41 |  | Tiahura | 6 | 90 | 27/11/2020 |
| 42 |  | Piha’ena | 2 | 34 | 09/10/2020 |
| 43 |  | Piha’ena | 3 | 79 | 30/10/2020 |
| 44 | Seychelles | FairyLand | 1 | 77 | 05/04/2022 |
| 45 |  | FairyLand | 2 | 65 | 05/04/2022 |
| 46 |  | FairyLand | 3 | 61 | 05/04/2022 |
| 47 |  | FairyLand | 4 | 59 | 06/04/2022 |
| 48 |  | FairyLand | 5 | 62 | 06/04/2022 |
| 49 |  | FairyLand | 6 | 66 | 06/04/2022 |
| 50 |  | FairyLand | 7 | 66 | 06/04/2022 |
| 51 |  | FairyLand | 8 | 63 | 08/04/2022 |
| 52 |  | FairyLand | 9 | 67 | 08/04/2022 |
| 53 |  | FairyLand | 10 | 60 | 08/04/2022 |
| 54 |  | FairyLand | 11 | 29 | 08/04/2022 |
| 55 |  | FairyLand | 12 | 61 | 08/04/2022 |
| 56 |  | Sunset | 1 | 61 | 12/04/2022 |
| 57 |  | Sunset | 2 | 66 | 12/04/2022 |
| 58 |  | Sunset | 3 | 57 | 12/04/2022 |
| 59 |  | FairyLand | 13 | 71 | 13/04/2022 |
| 60 |  | FairyLand | 14 | 70 | 13/04/2022 |
| 61 |  | Sunset | 1 | 64 | 27/03/2022 |
| 62 |  | Sunset | 2 | 62 | 27/03/2022 |
| 63 | Philippines | Dauin | 1 | 173 | 07/07/2022 |
| 64 |  | Dauin | 2 | 179 | 07/07/2022 |
