# Supplementary material for: Same calls, different meanings: Acoustic communication of Holocentridae
Source: PLoS One. 2024 Nov 21;19(11):e0312191. doi: 10.1371/journal.pone.0312191 (PMC11581312; doi:10.1371/journal.pone.0312191)
Supplement: S2 Table — Acc = acceleration, Chase_cs = conspecific chase, Chase_hs = heterospecific chase, Cp = competition, BC = broadcasting, BQ = body quivering. Underlined species correspond to those selected to perform the statistical analyses. For each species, behaviours whose the number acoustical events was < 5 were excluded from the analyses. Two additional events were removed from the Cp behaviour in N. sammara since they were extremely long with respect to the others. (DOCX) [file pone.0312191.s012.docx]

| Species | Acc | Chase_cs | Chase_hs | Cp | BC | BQ | Total per species |
| --- | --- | --- | --- | --- | --- | --- | --- |
| *M. berndti* | 33 162 | 82 346 | 17 52 | 2 20 | 26 59 | 5 18 | **165 657** |
| *M. kuntee* | 65 155 | 93 279 | 27 82 | 0 0 | 30 59 | 1 1 | **216 576** |
| *M. violacea* | 142 280 | 218 724 | 64 214 | 4 21 | 62 122 | 9 15 | **499 1376** |
| *N. argenteus* | 59 102 | 30 60 | 36 94 | 13 69 | 13 31 | 2 3 | **153 359** |
| *N. diadema* | 27 35 | 6 80 | 26 212 | 2 53 | 3 5 | 0 0 | **64 385** |
| *N. microstoma* | 9 19 | 22 121 | 35 95 | 1 5 | 2 2 | 1 2 | **70 244** |
| *N. sammara* | 33 75 | 96 298 | 177 522 | 73 576 | 23 39 | 0 0 | **402 1510** |
| *S. seychellense* | 27 57 | 31 103 | 21 65 | 2 6 | 21 34 | 0 0 | **102 265** |
| *S. spiniferum* | 18 45 | 9 42 | 68 289 | 2 2 | 18 31 | 0 0 | **115 409** |
| Total per behaviour | **413 930** | **587 2053** | **471 1625** | **99 752** | **198 382** | **19 39** | **1786 5781** |
| Total per behaviour for the selected species | **312 647** | **453 1526** | **383 1384** | **71 488** | **154 285** | **9 15** | **1382 4345** |
