# Supplementary material for: Same calls, different meanings: Acoustic communication of Holocentridae
Source: PLoS One. 2024 Nov 21;19(11):e0312191. doi: 10.1371/journal.pone.0312191 (PMC11581312; doi:10.1371/journal.pone.0312191)
Supplement: S3 Table — For each species, sound types whose the number of observations was < 5 were excluded from the statistical comparisons between behaviours. n = number total of sounds. (DOCX) [file pone.0312191.s013.docx]

| Species | Behaviour | n | | T1 | T2 | T3 | T3a | T3b | T3c |
| --- | --- | --- | --- | --- | --- | --- | --- | --- | --- |
| *M. kuntee* | Acc | 155 | | 130 83.9 | 17 11 | 8 5.2 | 0 0 | 8 5.2 | 0 0 |
|  | Chase_cs | 279 | | 193 69.2 | 49 17.6 | 37 13.3 | 0 0 | 37 13.3 | 0 0 |
|  | Chase_hs | 82 | | 64 78 | 11 13.4 | 7 8.5 | 0 0 | 7 8.5 | 0 0 |
|  | BC | 59 | | 41 69.5 | 14 23.7 | 4 6.8 | 0 0 | 4 6.8 | 0 0 |
| *M. violacea* | Acc | 280 | | 211 75.4 | 14 5 | 55 19.6 | 2 0.7 | 32 11.4 | 21 7.5 |
|  | Chase_cs | 724 | | 607 83.8 | 54 7.5 | 63 8.7 | 1 0.1 | 45 6.2 | 17 2.3 |
|  | Chase_hs | 214 | | 172 80.4 | 23 10.7 | 19 8.9 | 0 0 | 13 6.1 | 6 2.8 |
|  | BC | 122 | | 118 96.7 | 0 0 | 4 3.3 | 0 0 | 1 0.8 | 3 2.5 |
|  | BQ | 15 | | 12 80 | 1 6.7 | 2 13.3 | 0 0 | 2 13.3 | 0 0 |
| *N. diadema* | Acc | 35 | | 19 54.3 | 9 25.7 | 7 20 | 0 0 | 4 11.4 | 3 8.6 |
|  | Chase_cs | 80 | | 80 100 | 0 0 | 0 0 | 0 0 | 0 0 | 0 0 |
|  | Chase_hs | 212 | | 205 96.7 | 4 1.9 | 3 1.4 | 2 0.9 | 0 0 | 1 0.5 |
| *N. sammara* | Acc | 75 | | 65 86.7 | 0 0 | 10 13.3 | 0 0 | 2 2.7 | 8 10.7 |
|  | Chase_cs | 298 | | 285 95.6 | 5 1.7 | 8 2.7 | 1 0.3 | 5 1.7 | 2 0.7 |
|  | Chase_hs | 522 | | 486 93.1 | 12 2.3 | 24 4.6 | 1 0.2 | 10 1.9 | 13 2.5 |
|  | Cp | 488 | | 475 97.3 | 9 1.8 | 4 0.8 | 2 0.4 | 1 0.2 | 1 0.2 |
|  | BC | 39 | | 36 92.3 | 0 0 | 3 7.7 | 0 0 | 0 0 | 3 7.7 |
| *S. seychellense* | Acc | 57 | | 51 89.5 | 4 7 | 2 3.5 | 0 0 | 2 3.5 | 0 0 |
|  | Chase_cs | 103 | | 100 97.1 | 0 0 | 3 2.9 | 0 0 | 3 2.9 | 0 0 |
|  | Chase_hs | 65 | | 63 96.9 | 1 1.5 | 1 1.5 | 0 0 | 1 1.5 | 0 0 |
|  | BC | 34 | | 32 94.1 | 0 0 | 2 5.9 | 0 0 | 2 5.9 | 0 0 |
| Species | Behaviour | n | | T1 | T2 | T3 | T3a | T3b | T3c |
| *S. spiniferum* | Acc | 45 | | 26 57.8 | 14 31.1 | 5 11.1 | 0 0 | 3 6.7 | 2 4.4 |
|  | Chase_cs | 42 | | 36 85.7 | 0 0 | 6 14.3 | 0 0 | 0 0 | 6 14.3 |
|  | Chase_hs | 289 | | 256 88.6 | 16 5.5 | 17 5.9 | 1 0.3 | 9 3.1 | 7 2.4 |
|  | BC | 31 | | 23 74.2 | 5 16.1 | 3 9.7 | 0 0 | 2 6.5 | 1 3.2 |
| Number total of sounds / sound type | | | **4345** | **3786** | **262** | **297** | **10** | **193** | **94** |
| Number total of sounds / sound type used for comparison analyses between behaviours | | | **4282** | **3786** | **252** | **244** | **0** | **166** | **78** |
