# Supplementary material for: Same calls, different meanings: Acoustic communication of Holocentridae
Source: PLoS One. 2024 Nov 21;19(11):e0312191. doi: 10.1371/journal.pone.0312191 (PMC11581312; doi:10.1371/journal.pone.0312191)
Supplement: S4 Table — Significance level = 0.05. NS = non-significant. P values in bold are significant. DuE = event duration, Nsounds = number of sounds, rhyt = rhythm. (DOCX) [file pone.0312191.s014.docx]

| *M. kuntee* | χ^2^ | df | *P* |
| --- | --- | --- | --- |
| DuE | 8.49 | 3 | **0.037** |
| Nsounds | 9 | 3 | **0.029** |
| Rhyt | 2.57 | 3 | NS |
| *M. violacea* | **χ^2^** | **df** | ***P*** |
| DuE | 54.15 | 4 | **0** |
| Nsounds | 53.96 | 4 | **0** |
| Rhyt | 7.99 | 4 | NS |
| *N. diadema* | **χ^2^** | **df** | ***P*** |
| DuE | 33.64 | 2 | **0** |
| Nsounds | 36.52 | 2 | **0** |
| Rhyt | 3.43 | 2 | NS |
| *N. sammara* | **χ^2^** | **df** | ***P*** |
| DuE | 73.19 | 4 | **0** |
| Nsounds | 74.77 | 4 | **0** |
| Rhyt | 5.42 | 4 | NS |
| *S. seychellense* | **χ^2^** | **df** | ***P*** |
| DuE | 3.96 | 3 | NS |
| Nsounds | 4.98 | 3 | NS |
| Rhyt | 0.19 | 3 | NS |
| *S. spiniferum* | **χ^2^** | **df** | ***P*** |
| Nsounds | 14.91 | 3 | **0.002** |
