# Supplementary material for: Same calls, different meanings: Acoustic communication of Holocentridae
Source: PLoS One. 2024 Nov 21;19(11):e0312191. doi: 10.1371/journal.pone.0312191 (PMC11581312; doi:10.1371/journal.pone.0312191)
Supplement: S5 Table — Significance level = α = 0.05. Significance threshold of the Dunn test (dunn.test function with parameter ‘altp’ = FALSE) = α/2 = 0.025. NS = non-significant. P values in bold are significant. (DOCX) [file pone.0312191.s015.docx]

| *N. sammara* - DuE | Acc | Chase_cs | Chase_hs | Cp |
| --- | --- | --- | --- | --- |
| Chase_cs | NS |  |  |  |
| Chase_hs | NS | NS |  |  |
| Cp | **0.000** | **0.000** | **0.000** |  |
| BC | NS | **0.007** | **0.007** | **0.000** |
| *N. sammara* - Nsounds | Acc | Chase_cs | Chase_hs | Cp |
| Chase_cs | **0.022** |  |  |  |
| Chase_hs | NS | NS |  |  |
| Cp | **0.000** | **0.000** | **0.000** |  |
| BC | NS | **0.007** | **0.001** | **0.000** |
| *M. violacea* – DuE | Acc | Chase_cs | Chase_hs | BC |
| Chase_cs | **0.000** |  |  |  |
| Chase_hs | **0.000** | NS |  |  |
| BC | NS | **0.000** | **0.000** |  |
| BQ | NS | **0.015** | **0.014** | NS |
| *M. violacea* – Nsounds | Acc | Chase_cs | Chase_hs | BC |
| Chase_cs | **0.000** |  |  |  |
| Chase_hs | **0.000** | NS |  |  |
| BC | NS | **0.000** | **0.000** |  |
| BQ | NS | NS | **0.020** | NS |
| *M. kuntee* - DuE | Acc | Chase_cs | Chase_hs |  |
| Chase_cs | NS |  |  |  |
| Chase_hs | NS | NS |  |  |
| BC | NS | NS | NS |  |
| *M. kuntee* - Nsounds | Acc | Chase_cs | Chase_hs |  |
| Chase_cs | NS |  |  |  |
| Chase_hs | NS | NS |  |  |
| BC | NS | NS | NS |  |
| *S. spiniferum* - Nsounds | Acc | Chase_cs | Chase_hs |  |
| Chase_cs | NS |  |  |  |
| Chase_hs | NS | NS |  |  |
| BC | NS | NS | **0.001** |  |
| *N. diadema* - DuE | Acc | Chase_cs |  |  |
| Chase_cs | **0.000** |  |  |  |
| Chase_hs | **0.000** | NS |  |  |
| *N. diadema* - Nsounds | Acc | Chase_cs |  |  |
| Chase_cs | **0.000** |  |  |  |
| Chase_hs | **0.000** | NS |  |  |
