# Supplementary material for: Same calls, different meanings: Acoustic communication of Holocentridae
Source: PLoS One. 2024 Nov 21;19(11):e0312191. doi: 10.1371/journal.pone.0312191 (PMC11581312; doi:10.1371/journal.pone.0312191)
Supplement: S6 Table — Significance level = 0.05. P values in bold are significant. (DOCX) [file pone.0312191.s016.docx]

| Variables | F | *df* | *P* |
| --- | --- | --- | --- |
| DuE | 2.85 | 3 | **0.041** |
| Rhyt | 10.96 | 3 | **0.000** |
