# Supplementary material for: Same calls, different meanings: Acoustic communication of Holocentridae
Source: PLoS One. 2024 Nov 21;19(11):e0312191. doi: 10.1371/journal.pone.0312191 (PMC11581312; doi:10.1371/journal.pone.0312191)
Supplement: S7 Table — Significance level = 0.05. NS = non-significant. P values in bold are significant. (DOCX) [file pone.0312191.s017.docx]

| *S. spiniferum* - DuE | Acc | Chase_cs | Chase_hs |
| --- | --- | --- | --- |
| Chase_cs | NS |  |  |
| Chase_hs | NS | NS |  |
| BC | NS | NS | NS |
| *S. spiniferum* - Rhyt | Acc | Chase_cs | Chase_hs |
| Chase_cs | **0.003** |  |  |
| Chase_hs | NS | **0.000** |  |
| BC | NS | NS | NS |
