# Supplementary material for: Same calls, different meanings: Acoustic communication of Holocentridae
Source: PLoS One. 2024 Nov 21;19(11):e0312191. doi: 10.1371/journal.pone.0312191 (PMC11581312; doi:10.1371/journal.pone.0312191)
Supplement: S8 Table — (DOCX) [file pone.0312191.s018.docx]

| Species | Acc | Chase_cs | Chase_hs | Cp | BC | BD |
| --- | --- | --- | --- | --- | --- | --- |
| *M. kuntee* | 20 (65) | 42 (93) | 18 (27) | - | 15 (30) | - |
| *M. violacea* | 24 (142) | 56 (218) | 28 (64) | - | 9 (62) | 5 (9) |
| *N. diadema* | 7 (27) | 6 (6) | 17 (26) | - | - | - |
| *N. sammara* | 9 (33) | 20 (96) | 27 (177) | 25 (71) | 4 (23) | - |
| *S. seychellense* | 8 (27) | 9 (31) | 7 (21) | - | 5 (21) | - |
| *S. spiniferum* | 12 (18) | 7 (9) | 26 (68) | - | 6 (18) | - |
