# Supplementary material for: Same calls, different meanings: Acoustic communication of Holocentridae
Source: PLoS One. 2024 Nov 21;19(11):e0312191. doi: 10.1371/journal.pone.0312191 (PMC11581312; doi:10.1371/journal.pone.0312191)
Supplement: S9 Table — N = number of acoustic events. (DOCX) [file pone.0312191.s019.docx]

| Species | Behaviour | N | T1 |
| --- | --- | --- | --- |
| *M. kuntee* | Acc | 65 | 55 84.6 |
|  | Chase_cs | 93 | 63 67.7 |
|  | Chase_hs | 27 | 21 77.8 |
|  | BC | 30 | 19 63.3 |
| *M. violacea* | Acc | 142 | 116 81.7 |
|  | Chase_cs | 218 | 184 84.4 |
|  | Chase_hs | 64 | 48 75 |
|  | BC | 62 | 61 98.4 |
|  | BQ | 9 | 7 77.8 |
| *N. diadema* | Acc | 27 | 16 59.3 |
|  | Chase_cs | 6 | 6 100 |
|  | Chase_hs | 26 | 24 92.3 |
| *N. sammara* | Acc | 33 | 26 78.8 |
|  | Chase_cs | 96 | 91 94.8 |
|  | Chase_hs | 177 | 163 92.1 |
|  | Cp | 71 | 69 97.2 |
|  | BC | 23 | 20 87 |
| *S. seychellense* | Acc | 27 | 24 88.9 |
|  | Chase_cs | 31 | 31 100 |
|  | Chase_hs | 21 | 20 95.2 |
|  | BC | 21 | 19 90.5 |
| *S. spiniferum* | Acc | 18 | 10 55.6 |
|  | Chase_cs | 9 | 8 88.9 |
|  | Chase_hs | 68 | 63 92.6 |
|  | BC | 18 | 12 66.7 |
