# Supplementary material for: Same calls, different meanings: Acoustic communication of Holocentridae
Source: PLoS One. 2024 Nov 21;19(11):e0312191. doi: 10.1371/journal.pone.0312191 (PMC11581312; doi:10.1371/journal.pone.0312191)
Supplement: S10 Table — Significance level = 0.05. NS = non-significant. P values in bold are significant. Du = sound duration, fpeak = dominant frequency. (DOCX) [file pone.0312191.s020.docx]

| Species | Variable | χ^2^ | *df* | *P* |
| --- | --- | --- | --- | --- |
| *N. diadema* | Du | 22.27 | 2 | **0** |
|  | Fpeak | 5.12 | 2 | NS |
| *M. kuntee* | Du | 0.74 | 3 | NS |
|  | Fpeak | 12.12 | 3 | **0.007** |
| *N. sammara* | Du | 3.59 | 4 | NS |
|  | Fpeak | 11.7 | 4 | **0.02** |
| *S. seychellense* | Du | 5.59 | 3 | NS |
|  | Fpeak | 8.71 | 3 | **0.033** |
| *S. spiniferum* | Du | 13.21 | 3 | **0.004** |
|  | Fpeak | 24.98 | 3 | **0** |
| *M. violacea* | Du | 42.85 | 4 | **0** |
|  | Fpeak | 132.57 | 4 | **0** |
