# Supplementary material for: Same calls, different meanings: Acoustic communication of Holocentridae
Source: PLoS One. 2024 Nov 21;19(11):e0312191. doi: 10.1371/journal.pone.0312191 (PMC11581312; doi:10.1371/journal.pone.0312191)
Supplement: S11 Table — Significance level = α = 0.05. Significance threshold of the Dunn test (dunn.test function with parameter ‘altp’ = FALSE) = α/2 = 0.025. NS = non-significant. P values in bold are significant. Du = sound duration, fpeak = dominant frequency. (DOCX) [file pone.0312191.s021.docx]

| *N. diadema* - Du | Acc | Chase_cs |  |  |
| --- | --- | --- | --- | --- |
| Chase_cs | **0.014** |  |  |  |
| Chase_hs | **0.000** | **0.002** |  |  |
| *M. kuntee* - Fpeak | Acc | Chase_cs | Chase_hs |  |
| Chase_cs | **0.017** |  |  |  |
| Chase_hs | NS | NS |  |  |
| BC | NS | **0.010** | NS |  |
| *N. sammara* - Fpeak | Acc | Chase_cs | Chase_hs | Cp |
| Chase_cs | NS |  |  |  |
| Chase_hs | NS | NS |  |  |
| Cp | NS | **0.014** | **0.024** |  |
| BC | NS | NS | NS | NS |
| *S. seychellense* - Fpeak | Acc | Chase_cs | Chase_hs |  |
| Chase_cs | NS |  |  |  |
| Chase_hs | NS | NS |  |  |
| BC | NS | **0.024** | **0.017** |  |
| *S. spiniferum* - Du | Acc | Chase_cs | Chase_hs |  |
| Chase_cs | **0.022** |  |  |  |
| Chase_hs | NS | **0.001** |  |  |
| BC | NS | NS | NS |  |
| *S. spiniferum* - Fpeak | Acc | Chase_cs | Chase_hs |  |
| Chase_cs | NS |  |  |  |
| Chase_hs | NS | NS |  |  |
| BC | **0.007** | **0.000** | **0.000** |  |
| *M. violacea* - Du | Acc | Chase_cs | Chase_hs | BC |
| Chase_cs | **0.000** |  |  |  |
| Chase_hs | NS | **0.000** |  |  |
| BC | NS | **0.001** | NS |  |
| BQ | NS | NS | NS | NS |
| *M. violacea* - Fpeak | Acc | Chase_cs | Chase_hs | BC |
| Chase_cs | **0.000** |  |  |  |
| Chase_hs | **0.002** | **0.000** |  |  |
| BC | **0.019** | **0.000** | **0.000** |  |
| BQ | NS | 0.004 | NS | NS |
