# Supplementary material for: Same calls, different meanings: Acoustic communication of Holocentridae
Source: PLoS One. 2024 Nov 21;19(11):e0312191. doi: 10.1371/journal.pone.0312191 (PMC11581312; doi:10.1371/journal.pone.0312191)
Supplement: S13 Table — Significance level = 0.05. NS = non-significant. P values in bold are significant. (DOCX) [file pone.0312191.s023.docx]

| ***M. kuntee* - Duper** | **Acc** | **Chase_cs** | **Chase_hs** |
| --- | --- | --- | --- |
| Chase_cs | **0.014** |  |  |
| Chase_hs | NS | NS |  |
| BC | NS | NS | NS |
