# Supplementary material for: Same calls, different meanings: Acoustic communication of Holocentridae
Source: PLoS One. 2024 Nov 21;19(11):e0312191. doi: 10.1371/journal.pone.0312191 (PMC11581312; doi:10.1371/journal.pone.0312191)
Supplement: S14 Table — Significance level = 0.05. NS = non-significant. P values in bold are significant. Du = sound duration, npulses = number of pulses in sounds, F0 = fundamental frequency, lastpu = duration of the last pulse, fpeak = dominant frequency. (DOCX) [file pone.0312191.s024.docx]

| Species | Variable | χ^2^ | *df* | *P* |
| --- | --- | --- | --- | --- |
| *M. kuntee* | Du | 5.17 | 2 | NS |
|  | Npulses | 7.27 | 2 | **0.026** |
|  | F0 | 2.19 | 2 | NS |
|  | Lastpu | 1.3 | 2 | NS |
|  | Fpeak | 1.01 | 2 | NS |
| *M. violacea* | Du | 10.18 | 2 | **0.006** |
|  | Npulses | 13.4 | 2 | **0.001** |
|  | Lastpu | 1.5 | 2 | NS |
|  | Fpeak | 1.68 | 2 | NS |
