# Supplementary material for: Same calls, different meanings: Acoustic communication of Holocentridae
Source: PLoS One. 2024 Nov 21;19(11):e0312191. doi: 10.1371/journal.pone.0312191 (PMC11581312; doi:10.1371/journal.pone.0312191)
Supplement: S16 Table — Significance level = 0.05. NS = non-significant. Duper = pulse period, F0 = fundamental frequency. (DOCX) [file pone.0312191.s026.docx]

| ***M. kuntee*** | **F** | **df** | ***P*** |
| --- | --- | --- | --- |
| Duper | 0.68 | 2 | NS |
| ***M. violacea*** | **F** | **df** | ***P*** |
| F0 | 0.13 | 2 | NS |
| Duper | 1.57 | 2 | NS |
