# Supplementary material for: Same calls, different meanings: Acoustic communication of Holocentridae
Source: PLoS One. 2024 Nov 21;19(11):e0312191. doi: 10.1371/journal.pone.0312191 (PMC11581312; doi:10.1371/journal.pone.0312191)
Supplement: S17 Table — Significance level = 0.05. NS = non-significant. P values in bold are significant. Fpeak = dominant frequency, lastpu = duration of the last pulse. (DOCX) [file pone.0312191.s027.docx]

| Species | Variable | χ^2^ | *df* | *P* |
| --- | --- | --- | --- | --- |
| *M. kuntee* | Fpeak | 5.99 | 3 | NS |
| *N. sammara* | Lastpu | 1.14 | 2 | NS |
|  | Fpeak | 2.45 | 2 | NS |
| *S. spiniferum* | Fpeak | 2.08 | 2 | NS |
|  | Duper | 0.67 | 2 | NS |
| *M. violacea* | Lastpu | 1.94 | 2 | NS |
|  | Fpeak | 8.64 | 2 | **0.013** |
|  | Duper | 0.88 | 2 | NS |
