# Supplementary material for: Same calls, different meanings: Acoustic communication of Holocentridae
Source: PLoS One. 2024 Nov 21;19(11):e0312191. doi: 10.1371/journal.pone.0312191 (PMC11581312; doi:10.1371/journal.pone.0312191)
Supplement: S18 Table — Significance level = α = 0.05. Significance threshold of the Dunn test (dunn.test function with parameter ‘altp’ = FALSE) = α/2 = 0.025. NS = non-significant. P values in bold are significant. (DOCX) [file pone.0312191.s028.docx]

| ***M. violacea* - Fpeak** | Acc | Chase_cs |
| --- | --- | --- |
| Chase_cs | **0.009** |  |
| Chase_hs | **0.007** | NS |
