# Supplementary material for: Same calls, different meanings: Acoustic communication of Holocentridae
Source: PLoS One. 2024 Nov 21;19(11):e0312191. doi: 10.1371/journal.pone.0312191 (PMC11581312; doi:10.1371/journal.pone.0312191)
Supplement: S19 Table — Significance level = 0.05. NS = non-significant. P values in bold are significant. Du = sound duration, npulses = number of pulses in sounds, lastpu = duration of the last pulse, F0 = fundamental frequency, fpeak = dominant frequency, duper = pulse period. (DOCX) [file pone.0312191.s029.docx]

| Variable | χ^2^ | *df* | *P* |
| --- | --- | --- | --- |
| Du | 1.91 | 2 | NS |
| Npulses | 4.95 | 2 | NS |
| F0 | 6.7 | 2 | **0.035** |
| Lastpu | 12.81 | 2 | **0.002** |
| Fpeak | 3.65 | 2 | NS |
| Duper | 0.72 | 2 | NS |
