# Supplementary material for: Same calls, different meanings: Acoustic communication of Holocentridae
Source: PLoS One. 2024 Nov 21;19(11):e0312191. doi: 10.1371/journal.pone.0312191 (PMC11581312; doi:10.1371/journal.pone.0312191)
Supplement: S21 Table — Significance level = 0.05. NS = non-significant. P values in bold are significant. Du = sound duration, lastpu = duration of the last pulse, F0 = fundamental frequency, fpeak = dominant frequency, duper = pulse period. (DOCX) [file pone.0312191.s031.docx]

| ***N. sammara*** | **t** | **df** | ***P*** |
| --- | --- | --- | --- |
| Du | -2.01 | 19 | NS |
| Lastpu | -0.71 | 19 | NS |
| F0 | -0.67 | 9 | NS |
| Fpeak | -1.29 | 19 | NS |
| Duper | -0.20 | 19 | NS |
| ***S. spiniferum*** | **t** | **df** | ***P*** |
| Du | -0.15 | 11 | NS |
| Lastpu | -2.28 | 11 | **0.043** |
| F0 | -2.10 | 9 | NS |
| Duper | 1.58 | 11 | NS |
