# Supplementary material for: Same calls, different meanings: Acoustic communication of Holocentridae
Source: PLoS One. 2024 Nov 21;19(11):e0312191. doi: 10.1371/journal.pone.0312191 (PMC11581312; doi:10.1371/journal.pone.0312191)
Supplement: S22 Table — Significance level = 0.05. NS = non-significant. P values in bold are significant. Du = sound duration, fpeak = dominant frequency, lastpu = duration of the last pulse, duper = pulse period. (DOCX) [file pone.0312191.s032.docx]

| ***N. sammara*** | **t** | **df** | ***P*** |
| --- | --- | --- | --- |
| Du | 0.40 | 13 | NS |
| Fpeak | -2.02 | 13 | NS |
| Lastpu | 1.81 | 13 | NS |
| Duper | 2.24 | 13 | **0.043** |
