# Supplementary material for: Same calls, different meanings: Acoustic communication of Holocentridae
Source: PLoS One. 2024 Nov 21;19(11):e0312191. doi: 10.1371/journal.pone.0312191 (PMC11581312; doi:10.1371/journal.pone.0312191)
Supplement: S24 Table — Significance level = 0.05. NS = non-significant. Npulses = number of pulses in sounds, fpeak = dominant frequency. (DOCX) [file pone.0312191.s034.docx]

| ***N. sammara*** | **W** | ***P*** |
| --- | --- | --- |
| Npulses | 30 | NS |
| ***S. spiniferum*** | **W** | ***P*** |
| Npulses | 24.5 | NS |
| Fpeak | 12 | NS |
