# Supplementary material for: Same calls, different meanings: Acoustic communication of Holocentridae
Source: PLoS One. 2024 Nov 21;19(11):e0312191. doi: 10.1371/journal.pone.0312191 (PMC11581312; doi:10.1371/journal.pone.0312191)
Supplement: S25 Table — Significance level = 0.05. NS = non-significant. (DOCX) [file pone.0312191.s035.docx]

| ***S. spiniferum*** | **t** | **df** | ***P*** |
| --- | --- | --- | --- |
| Lastpu | 1.58 | 334 | NS |
| Duper | -1.05 | 334 | NS |
