# Supplementary material for: Same calls, different meanings: Acoustic communication of Holocentridae
Source: PLoS One. 2024 Nov 21;19(11):e0312191. doi: 10.1371/journal.pone.0312191 (PMC11581312; doi:10.1371/journal.pone.0312191)
Supplement: S26 Table — Significance level = 0.05. NS = non-significant. P values in bold are significant. Du = sound duration, npulses = number of pulses in sounds, F0 = fundamental frequency, fpeak = dominant frequency, lastpu = duration of the last pulse, duper = pulse period. (DOCX) [file pone.0312191.s036.docx]

| ***S. spiniferum*** | **W** | ***P*** |
| --- | --- | --- |
| Du | 2671 | NS |
| Npulses | 2323 | NS |
| F0 | 692.5 | **0.001** |
| Fpeak | 3047 | **0.006** |
| ***N. sammara*** | **W** | ***P*** |
| Du | 5015.5 | NS |
| Npulses | 5972.5 | **0.034** |
| F0 | 3329 | **0.036** |
| Fpeak | 7269.5 | **0.000** |
| Lastpu | 6542 | **0.003** |
| Duper | 2550 | **0.000** |
| ***M. violacea*** | **W** | ***P*** |
| Du | 6687 | **0.000** |
| Npulses | 6939 | **0.000** |
| F0 | 1483 | NS |
| Fpeak | 5632.5 | **0.000** |
| Lastpu | 3153.5 | **0.032** |
| Duper | 2490 | **0.000** |
